# Landmark Models for Optimizing the Use of Repeated Measurements of Risk Factors in Electronic Health Records to Predict Future Disease Risk

**DOI:** 10.1093/aje/kwy018

**Published:** 2018-03-23

**Authors:** Ellie Paige, Jessica Barrett, David Stevens, Ruth H Keogh, Michael J Sweeting, Irwin Nazareth, Irene Petersen, Angela M Wood

**Affiliations:** 1 Department of Public Health and Primary Care, School of Clinical Medicine, University of Cambridge, Cambridge, United Kingdom; 2 National Centre for Epidemiology and Population Health, Research School of Population, The Australian National University, Canberra, Australia; 3 MRC Biostatistics Unit, University of Cambridge, Cambridge, United Kingdom; 4 Department of Medical Statistics, London School of Hygiene and Tropical Medicine, London, United Kingdom; 5 Institute of Epidemiology and Health, Research Department of Primary Care and Population Health, Institute of Epidemiology and Health Care, University College London, London, United Kingdom

**Keywords:** cardiovascular disease, dynamic risk prediction, electronic health records, landmarking, mixed-effects models, primary care records

## Abstract

The benefits of using electronic health records (EHRs) for disease risk screening and personalized health-care decisions are being increasingly recognized. Here we present a computationally feasible statistical approach with which to address the methodological challenges involved in utilizing historical repeat measures of multiple risk factors recorded in EHRs to systematically identify patients at high risk of future disease. The approach is principally based on a 2-stage dynamic landmark model. The first stage estimates current risk factor values from all available historical repeat risk factor measurements via landmark-age–specific multivariate linear mixed-effects models with correlated random intercepts, which account for sporadically recorded repeat measures, unobserved data, and measurement errors. The second stage predicts future disease risk from a sex-stratified Cox proportional hazards model, with estimated current risk factor values from the first stage. We exemplify these methods by developing and validating a dynamic 10-year cardiovascular disease risk prediction model using primary-care EHRs for age, diabetes status, hypertension treatment, smoking status, systolic blood pressure, total cholesterol, and high-density lipoprotein cholesterol in 41,373 persons from 10 primary-care practices in England and Wales contributing to The Health Improvement Network (1997–2016). Using cross-validation, the model was well-calibrated (Brier score = 0.041, 95% confidence interval: 0.039, 0.042) and had good discrimination (C-index = 0.768, 95% confidence interval: 0.759, 0.777).

Using electronic health records (EHRs) to systematically identify persons at high risk of developing future disease outcomes has the potential to increase the cost-effectiveness of health care (1); however, existing risk prediction models do not fully optimize available historical data. The development of computationally feasible statistical methods for predicting future disease risk from existing EHRs presents specific methodological challenges and opportunities.

First, risk prediction models are typically developed using traditional prospective study designs, which define a baseline origin at which risk factors were observed and from which to predict future disease risk. However, EHRs are dynamic in nature—for example, in primary-care records, an individual’s follow-up begins at registration with a general practice, risk factors are measured sporadically during general practice visits, and follow-up continues until the person transfers out or dies. Defining arbitrary time origins for model development without allowing for the in- and outflow of study participants over time can introduce bias (2). Second, risk prediction models typically use single measures of error-prone risk factors (e.g., blood pressure and cholesterol), but EHRs often contain data on risk factors measured repeatedly over time which could be utilized both for model development and for predicting future disease risk. In particular, repeated measurements can be used to predict error-free “estimated current values” of risk factors, which may increase their predictive ability (3). Third, most risk prediction models require complete risk factor data in order to predict future risk. An exception in cardiovascular disease (CVD) risk prediction is the QRISK2 model (4), which has a built-in tool for substituting missing data on risk factors using age- and sex-specific population average values. Notably, this substitution approach is not compatible with the multiple-imputation approach used for model development of QRISK2 and has not yet been formally validated (5). Since EHR systems are primarily designed for patient management and administrative purposes, there can be large amounts of unobserved information on risk factors that needs to be handled appropriately and compatibly both in model development and for predicting future disease risk.

While multiple methods exist for developing risk prediction models using EHRs, a previous systematic review found that only 8% of studies modeled repeated longitudinal measures, only 54% accounted for missing data, only 16% appropriately accounted for censoring and loss to follow-up, and none assessed informative observations (where the clinic visit itself provides meaningful information) (6). Our aim was to establish a computationally feasible generic statistical framework that accounts for these potential advantages and biases of EHRs in the development of dynamic risk prediction models that leverage repeated measurements and handle unobserved data on routinely recorded risk factors. Our approach combines 2 existing methods, landmark-age models and multivariate linear mixed-effects models (2, 7). A landmark age is a reference point (e.g., 40, 45, 50,…, 85 years) at which we want to make risk predictions using risk factor information collected up to that age. A series of prediction models, which we call landmark-age models, are constructed with time origin at the landmark age and past risk factor information from eligible individuals (e.g., in our setting these are persons who are currently registered with a general practice and at future risk of disease at the landmark age). As such, individuals may contribute to one or more prediction models, depending on their eligibility at the landmark age reference points.

Typically, landmark-age models are constructed using Cox proportional hazards models with the last observed risk factor values. We propose an extension to this, whereby we replace the last observed values with error-free risk factor values estimated from a multivariate linear mixed-effects model using all available repeated measures of past risk factor values for each landmark age (8). Multivariate mixed-effects models intrinsically handle unobserved data and sporadically recorded repeat measures (9) and their measurement errors (10). The approach also provides flexibility to account for the number (or rate) of clinic visits as a proxy for illness severity or health anxiety. There is a strong body of statistical evidence showing the benefits and potential applications of modeling longitudinal data using mixed-effects linear regression models (3, 11–14), but this method is not often employed in the development of risk prediction models using EHRs (6). Moreover, using landmarking to model data in EHRs has been previously proposed (15) and has been combined with univariate mixed-effects modeling (16, 17) but not in the context of dynamic risk prediction models.

In the current study, we explore how landmarking can be combined with multivariate mixed-effects linear regression models to leverage the advantages of each method in order to generate dynamic risk prediction models suitable for use in EHRs. We illustrate our approach through the estimation of 10-year CVD risk using EHRs from 10 general practices in England and Wales.

## METHODS

### Data source

We used patient data from 10 randomly selected general practices that contributed data to The Health Improvement Network (18), a United Kingdom general practice database that derives data from routine administrative and clinical practice. During consultations with patients, family physicians enter data on medical symptoms and diagnoses using Read codes (19) (a hierarchical classification system), while information on drug prescriptions is entered automatically into the EHRs. The Health Improvement Network captures information on patient demographic characteristics, practice-level data, diagnoses and symptoms, specialist referrals, laboratory testing, disease monitoring, prescribing, and death. For this study, we created code lists for the risk factors and outcomes using previously described methods (20). Code lists were reviewed by a clinician (I.N.) and have been published on ClinicalCodes.org.

The main outcome was newly recorded diagnoses of nonfatal or fatal CVD, where CVD was defined, as with previous primary care risk scores (4), as angina, myocardial infarction, stroke, transient ischemic attack, or major coronary surgery and revascularization. Cause of death was ascertained using Read codes.

Risk factors were selected on the basis of those in the validated American College of Cardiology/American Heart Association Pooled Cohort Risk Assessment Equations (21, 22) and included age, sex, diabetes status (binary, ascertained using Read codes (23)), smoking status (binary), systolic blood pressure (SBP) (adjusted for hypertension treatment), total cholesterol level, and high-density lipoprotein cholesterol (HDL-C) level. Once an individual had a diabetes diagnosis or a prescription for a blood-pressure–lowering medication, he or she was considered to have this condition/treatment throughout follow-up. Values for SBP, total cholesterol, and HDL-C were standardized by centering on sex-specific means and dividing by the standard deviation.

### Study population

Data were available from January 1, 1997, to January 18, 2016. Individuals entered the study from the latest of the following dates: 1) the date of registration at a general practice plus 6 months; 2) the date for acceptable computer usage (quality measurement defined as the year in which a general practice continuously used their computer system for recording of medical events and prescribing) (24); 3) the date for acceptable mortality reporting (the date on which mortality recording reflected that of the United Kingdom general population) (25); 4) the date on which the individual turned 30 years of age; or 5) January 1, 1997. Individuals exited the study at the earliest of the following dates: 1) their first (i.e., “incident”) newly recorded CVD event; 2) transfer out of the general practice; 3) their date of death; or 4) January 18, 2016. The target population for which we wanted to estimate CVD risk included persons with general practice records and without a history of CVD or statin prescriptions (see Web Figure 1, available at https://academic.oup.com/aje). We excluded participants with statin prescriptions, as these individuals are already being treated for being at risk of developing CVD and as such would not need to be identified by a screening algorithm. In addition, the study sample excluded persons with unknown sex, persons with a study entry date after age 85 years, and persons with no measurements of smoking status, SBP, total cholesterol, or HDL-C between study entry and study exit (Web Figure 1).

The following measurements were considered biologically implausible and were changed to “missing” for the analysis: SBP <60 mm Hg or >250 mm Hg (26); total cholesterol level <1.75 mmol/L or >20 mmol/L (27); and HDL-C level <0.3 mmol/L or >3.1 mmol/L (26) (out of a total 1,675,241 measurements, 12,352 measurements were changed to missing).

The scheme under which The Health Improvement Network was to obtain and provide anonymized patient data was approved by the National Health Service South-East Multicenter Research Ethics Committee in 2002, and scientific approval to undertake this study was obtained from the IQVIA World Publications Scientific Review Committee (IQVIA, Durham, North Carolina). E.P., J.B., D.S., I.P., and A.M.W. had full access to the data used to create the study population. This article follows RECORD reporting guidelines (Web Table 1) (28).

### Statistical analysis

#### Two-stage dynamic risk prediction model

**Figure 1.**
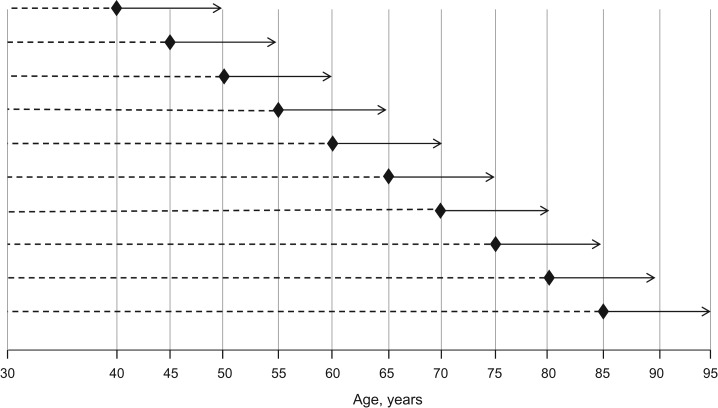
Schematic showing the landmark age approach. The dashed lines indicate historical repeat measures of smoking status, systolic blood pressure, total cholesterol, and high-density lipoprotein cholesterol, modeled by means of landmark-age–specific multivariate linear mixed-effects models. The diamonds show the landmark age (time of risk prediction). The arrows indicate the 10-year follow-up to the point of a cardiovascular disease event or censoring, modeled via a landmark Cox model.

We used a 2-stage approach to construct a dynamic risk prediction model, first modeling historical repeated risk factor measurements using multivariate mixed-effects linear models and then estimating 10-year CVD risk using Cox proportional hazards models (Figure 1). We briefly present the methods here and provide more detail in the Web Appendix. In both stages, models were developed at landmark ages (40, 45,…, 85 years) for eligible participants, defined as those 1) registered with a general practice at the landmark age, 2) with no CVD diagnoses prior to the landmark age, and 3) with no statin prescription prior to the landmark age. Treating each landmark age as a time origin, past risk factor information was extracted from age 30 years onwards and participants were followed up for 10 years until their first CVD event or the study exit date (Figure 1). Crude incidence rates by age at study entry, sex, and calendar year of statin prescription were calculated.

#### Estimation of error-free current risk factor values

For each landmark age and separately for males and females, we fitted multivariate mixed-effects linear regression models (9) on past repeat measurements for smoking status, SBP, total cholesterol, and HDL-C. Each model included fixed intercepts and slopes for each risk factor, a time-dependent covariate for initiation of blood-pressure–lowering medications for SBP, and correlated individual-specific random intercepts for all 4 risk factors. These models were estimable for persons with at least 1 measurement of at least 1 risk factor. From each model, we estimated the error-free *current risk factor values* (i.e., the predicted values at the landmark age) using the best linear unbiased predictors from the empirical Bayes posterior distribution of the random intercepts, conditional on the past observed risk factor measurements.

#### Estimating 10-year CVD risk

Ten-year CVD risk was estimated from a landmark age Cox proportional hazards model, stratified by sex and with time since landmark age as the underlying time variable. The model adjusted for landmark age and landmark age squared and included the following risk factors: last observed diabetes status; last observed treatment for hypertension; and estimated current risk factor values for smoking status, SBP, total cholesterol, and HDL-C. Participants were followed up for a maximum of 10 years. Therefore, proportional hazards are assumed only across a 10-year period. A “super-landmark model” approach (7) was used with robust standard errors. A super-landmark model is a version of landmarking in which the data sets contributing to the landmark models across all landmark ages are stacked and a single time-to-event model is fitted to the stacked data set (Web Appendix).

#### Assessment of predictive ability

The performance of the 10-year CVD risk predictions was assessed with measures of calibration (i.e., calibration plots by decile of predicted risk), predictive accuracy (i.e., Brier scores; an average of the squared difference between the observed outcome and predicted risk, where lower scores indicate better predictive accuracy and zero means perfect calibration), and discrimination (i.e., C-index; a measure of how well the model discriminates between persons with and without CVD (29, 30)). We estimated the C-index over all participants (calculated over pairs of different individuals) and also separately at each landmark age. The latter is estimated on subsets of persons of the same age; thus, we call this an age-adjusted C-index, which naturally will have lower values to reflect poorer discrimination (31). We used 10-fold cross-validation, splitting the data by general practice, to account for overoptimism.

The above 10-year CVD risk predictions were compared against predictions from 1) a “basic” landmark-age model, which included sex, age, last observed diabetes status, and last observed treatment for hypertension; 2) a dynamic landmark-age model with landmark age interactions with each covariate; 3) a dynamic landmark-age model with last observed measurements of all risk factors instead of estimated current risk factor values; and 4) a dynamic landmark-age model using cumulative mean values of all historical measurements recorded *before* each landmark age, of smoking status, SBP, total cholesterol, and HDL-C. Predictions from models 3 and 4 were only estimable for persons with 1 or more measurements of *all* risk factors, which we call the restricted sample.

#### Sensitivity analyses

We conducted 4 sensitivity analyses. First, instead of using all available historical repeat measurements of risk factors, we restricted the data to be within 10 years before each landmark age. Second, we adjusted the results of the multivariate mixed-effects models for the annual rate of repeated measurements in the 5 years before each landmark age (as a proxy to account for bias due to sicker or more health-conscious individuals’ having more repeats (32)). Third, instead of estimating current risk factor values from only past information, we estimated the future 10-year average risk factor levels from a multivariate mixed-effects model derived from both past and future risk factor information within the 10-year future horizon (Web Figure 2). Importantly, only past observed risk factors were subsequently used in the prediction of the future 10-year average risk factor levels for the Cox model. Fourth, since it might be useful to identify patients who are still at high absolute risk even after treatment with statins, we reran the main analyses including statin users in the models. The mixed-effects model including a time-dependent covariate for statin therapy initiation for total cholesterol and statin therapy at the landmark age was included as a risk factor in the Cox model.

All analyses were performed using Stata 14.2 (StataCorp LLC, College Station, Texas), and 95% confidence intervals were generated for all measures of association.

## RESULTS

### Study sample

The target population included 41,373 persons with general practice records and without a history of CVD or statin use at study entry. Of these individuals, 32,328 persons (78%) had at least 1 measurement of smoking status, SBP, total cholesterol, or HDL-C recorded before the first CVD event or statin prescription (Web Figure 1). Mean age at study entry was 47.9 (standard deviation, 13.6) years; 17,592 participants (54%) were male, and 5,617 (17%) were prescribed statins after study entry (Table 1). Participants generally had more repeat measures of SBP than of smoking status, total cholesterol, and HDL-C (Table 1). On average, there were 1.1 years between repeated measurements of smoking status, 0.5 years between repeated measurements of SBP, 1.1 years between repeated measurements of total cholesterol, and 1.2 years between repeated measurements of HDL-C.

**Table 1.** Characteristics of Participants in the Study Sample, The Health Improvement Network, United Kingdom, 1997–2016

Characteristic	Sample and Baseline Characteristic						Mean (SD) No. of Measurements per Year	
	Study Sample (*n* = 32,328)			Restricted Sample^a^ (*n* = 12,292)			Study Sample	Restricted Sample^a^
	No. of Persons	%	Mean (SD)	No. of Persons	%	Mean (SD)		
Age at study entry, years			47.9 (13.6)			47.5 (12.3)		
Male sex	17,592	54		6,819	55			
History of diabetes^b^	3,743	12		2,175	18			
Prescription for blood-pressure–lowering medication^b^	9,935	31		4,685	38			
Prescription for statins^b^	5,617	17		2,003	16			
Current smoker^b^	9,453	29		3,358	27		0.6 (0.4)	0.6 (0.4)
Systolic blood pressure, mm Hg^c^			134.8 (21.0)			135.3 (21.1)	1.4 (1.4)	1.6 (1.4)
Total cholesterol level, mmol/L^c^			5.5 (1.1)			5.4 (1.0)	0.4 (0.4)	0.5 (0.4)
HDL-C level, mmol/L^c^			1.4 (0.4)			1.4 (0.4)	0.3 (0.3)	0.4 (0.3)

Abbreviations: HDL-C, high-density lipoprotein cholesterol; SD, standard deviation.

^a^ The restricted sample contained only patients with at least 1 measurement for each variable (smoking status, systolic blood pressure, total cholesterol, and HDL-C).

^b^ Number and percentage were calculated across the follow-up period (e.g., a diagnosis of diabetes at any point during follow-up was counted as a history of diabetes for that individual).

^c^ Based on the first measurement taken after study entry.

Overall, 2,861 participants (7%) had a newly recorded CVD event over the course of a mean 10.4 (standard deviation, 5.6) years of follow-up. Crude CVD incidence rates per 1,000 person-years increased from 2.9 for persons aged 40–44 years to 35.2 for persons aged 80–84 years; rates were higher in men than in women, and they decreased among statin users by increasing calendar year (Table 2). Participants in the study sample and the restricted sample (*n* = 12,292 (30% of the target population); Web Figure 1) were similar in terms of age at study entry, sex, SBP, and total and HDL-C levels, but those in the restricted sample were more likely to have diabetes (Table 1). The study sample had more males than the target population but was otherwise similar (Web Table 2).

**Table 2.** Crude Cardiovascular Disease Incidence Rate per 1,000 Person-Years According to Age at Study Entry, Sex, and Calendar Year of Statin Prescription, The Health Improvement Network, United Kingdom. 1997–2016

Factor	No. of Incident CVD Cases	Total No. of PY	Crude IR per 1,000 PY
Age at study entry, years			
40–44	167	57,754	2.9
45–49	239	53,056	4.5
50–54	307	49,903	6.2
55–59	356	37,132	9.6
60–64	382	29,552	12.9
65–69	396	22,417	17.7
70–74	386	15,626	24.7
75–79	299	10,575	28.3
80–84	187	5,317	35.2
Sex			
Male	1,520	198,797	7.6
Female	1,341	232,166	5.8
Calendar year of statin initiation^a^			
1997–2001	225	4,828	46.6
2002–2006	968	38,857	24.9
2007–2011	687	46,662	14.7
2012–2016	365	27,543	13.3

Abbreviations: CVD, cardiovascular disease; IR, incidence rate; PY, person-years.

^a^ Calendar year of the prescribing date of the index statin prescription.

### Estimates from the landmark models

Regression coefficients from the age- and sex-specific multivariate linear mixed-effects models and hazard ratios for the Cox models, without 10-fold cross-validation, are provided in Web Tables 3–6. Overall, the values of the fixed intercepts from the multivariate mixed-effects linear models show that SBP and total cholesterol level increased over the landmark ages, whereas HDL-C and smoking status decreased (Web Table 3). In addition, hazard ratios were generally stronger for the model using estimated current risk factor values than for the model using the last observed values or cumulative mean values (Web Table 6).

### Assessment of 10-year CVD risk

**Figure 2.**
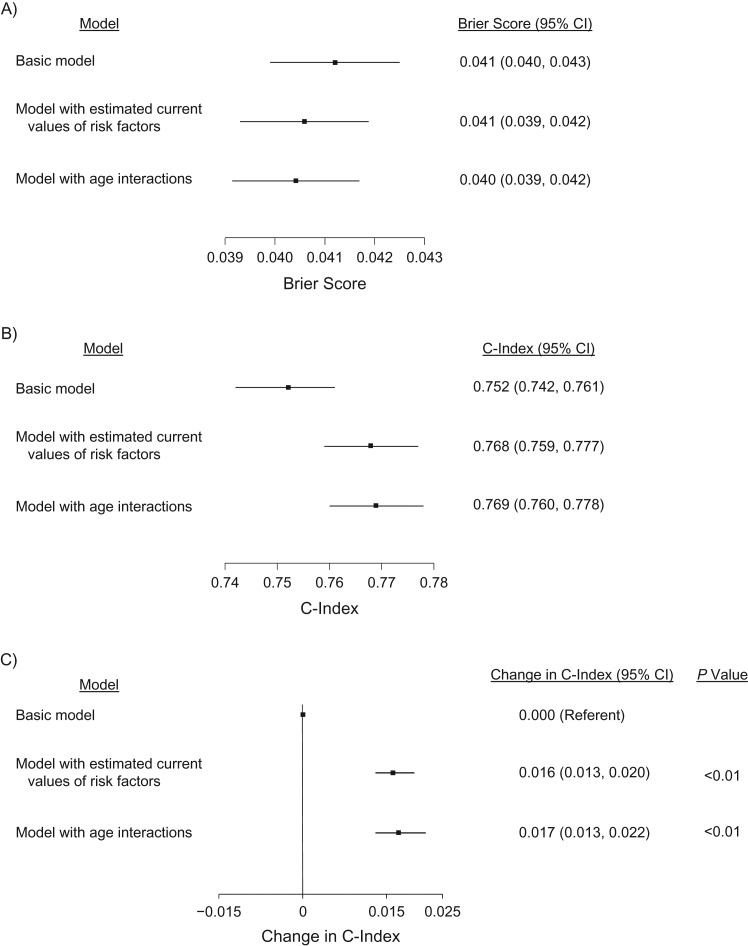
Calibration and risk discrimination statistics for 3 models of cardiovascular disease risk prediction (*n* = 32,328), The Health Improvement Network, United Kingdom, 1997–2016. A) Calibration statistics for each risk prediction model. The graph shows the Brier score (▪) and 95% confidence interval (CI; bars) for each model. A lower Brier score is interpreted as better calibration. B) Risk discrimination statistics for each risk prediction model. The graph shows the C-index (▪) and 95% CI (bars) for each model. A higher C-index value is interpreted as better discrimination. C) Change in risk discrimination for each risk prediction model. The graph shows the change in C-index (▪) and its 95% CI (bars) for each risk prediction model in relation to the basic model (referent). The basic model included age and sex plus the last observed measures for diabetes status and hypertension treatment. The model with estimated current values of the risk factors included all factors in the basic model plus predicted current values for smoking status, systolic blood pressure, total cholesterol, and high-density lipoprotein cholesterol. The model with age interactions included all factors in the basic model plus predicted current values for smoking status, systolic blood pressure, total cholesterol, and high-density lipoprotein cholesterol, plus interactions of age with all risk factors.

**Figure 3.**
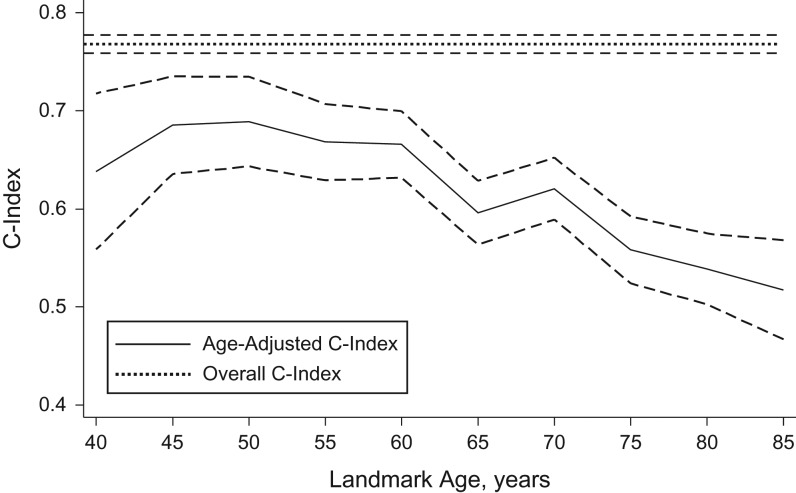
Overall and age-adjusted values for C-index, The Health Improvement Network, United Kingdom, 1997–2016. Dashed lines, 95% confidence intervals.

In the landmark model with estimated current risk factor values, 28% of individuals had an estimated 10-year CVD risk of ≥10%, and 10% had an estimated risk of ≥20%. The model appeared well-calibrated (Web Figure 3A), had a Brier score of 0.041 (95% confidence interval (CI): 0.039, 0.042) (Figure 2A), and had an overall C-index of 0.768 (95% CI: 0.759, 0.777) (Figure 2B). The C-index was improved by 0.016 (95% CI: 0.013, 0.020) in comparison with the basic model (Figure 2C). Discrimination was better at younger ages (Figure 3). Additional age interactions did not further improve calibration or risk discrimination (Web Figure 3B and Figure 2B). The basic model (including only age, diabetes status, and treatment for hypertension) also appeared well calibrated (Web Figure 3C), had a Brier score of 0.041 (95% CI: 0.040, 0.043) (Figure 2A), and had a lower overall C-index of 0.752 (95% CI: 0.742, 0.761) (Figure 2B). Similar to the main model, the basic model also discriminated risk better at younger ages than at older ages (Web Figure 4).

Estimated 10-year CVD risk appeared slightly higher in models using last observed and cumulative mean risk factor values as compared with estimated current values (Web Figure 5). Calibration, Brier scores, and C-indices were similar across the landmark models with last observed, cumulative mean, or estimated current risk factor values (Web Figures 6 and 7). Risk discrimination was better at younger ages than at older ages across all models (Web Figure 8).

### Sensitivity analyses

There was no difference in risk discrimination when the model was restricted to using historical repeated-measures data collected up to 10 years before the landmark age (C-index = 0.768, 95% CI: 0.758, 0.777) or when the estimated current risk factor values were adjusted for the rate of clinic visits (C-index = 0.766, 95% CI: 0.756, 0.775). However, we observed an increase in risk discrimination using estimated future 10-year average risk factor levels (C-index = 0.774, 95% CI: 0.765, 0.783) instead of estimated current risk factor values. C-indices were lower when statin users were included in the analysis, but the patterns of risk discrimination and calibration remained the same as in the main analysis (Web Tables 7 and 8).

## DISCUSSION

In this paper, we have presented a computationally feasible statistical framework for developing dynamic risk prediction models for use on EHRs with historical repeated measures of risk factors. The 2-stage landmark approach combines Cox proportional hazards regression and age-specific multivariate linear mixed-effects models, which account for sporadically recorded repeat measures, unobserved data, and measurement errors. We illustrated the framework for the derivation and validation of a primary-care dynamic risk prediction model for 10-year CVD risk, but it has potential for wider application to other diseases and conditions and for use on other electronic patient records in which repeated measurements are recorded, such as those collected in secondary-care settings.

Our motivation was based on optimizing electronic primary-care data for automatically identifying high-risk individuals for full formal disease risk assessment, rather like a prescreening tool with the potential to increase the cost-effectiveness of health care. For example, several international guidelines for CVD risk assessment and management (21, 33–35) recommend using a systematic strategy for prioritizing people for full formal risk assessment on the basis of an estimate of their CVD risk using risk factors already recorded in EHRs. CVD risk assessment tools, such as the Framingham risk model (36) and QRISK2 (4), are now integrated into electronic primary-care record systems, but they are not purposefully designed for prescreening use. The QRISK2 model estimates CVD risk using the last observed values for the numerous risk factors, and when data are missing, imputes them using age- and sex-specific population averages for continuous risk factors or assumes no adverse clinical indicators. Our proposed framework optimizes all available historical risk factor values, handling potential bias from spurious one-off measurements, and when data are missing, intrinsically imputes them using all other risk factor information. Future work should formally compare such models for prescreening use and assess their cost-effectiveness.

For illustration, we compared a basic CVD risk model using sex, age, diabetes status, and treatment for hypertension against extended risk models with additional risk factors incorporated as cumulative means, last observed values, or estimated current risk factor values for smoking status, SBP, total cholesterol, and HDL-C. Our findings showed a modest improvement in risk discrimination when including estimated current values of additional risk factors but no difference in risk discrimination in the restricted data set when comparing additional risk factors incorporated as last observed, cumulative means, or estimated current risk factor values. Cumulative mean risk factor values handle sporadically recorded repeat measurements and account for measurement errors, but they are only estimable for persons with at least 1 historical measurement on all risk factors and thus are not suitable for population-wide screening. A major strength of the landmark model with estimated current values of risk factors is that it can be applied to persons with at least 1 measure on any of the risk factors included in the multivariate mixed model (in our illustration, this was approximately 80% of individuals).

Another strength of our landmark framework is that it was developed and internally validated using data that reflected the complexity and messiness of the EHRs that would be used to estimate future disease risk for individuals, unlike risk prediction models developed using purpose-designed cohort studies. Importantly, the assumptions made about the dynamic nature of the historical repeat-measures data, unobserved risk factors, and measurement errors in the model development are compatible with the assumptions required for making a risk prediction for a new individual using data from EHRs.

In our sensitivity analysis, we investigated the use of predicted future 10-year average risk factor levels instead of estimated current values and observed a modest improvement in risk discrimination. This suggests that future risk factor values for smoking, SBP, total cholesterol, and HDL-C are more predictive of future 10-year CVD risk than current values. A considerable limitation in this analysis is that it ignores informative censoring of individuals due to death or CVD events in the multivariate mixed-effects model, although evidence from empirical and simulation studies (11, 14) suggests that there is often little to be gained from more complex modeling (e.g., joint models (37)).

Other methods with which to develop risk prediction models for use on EHRs exist, including machine learning approaches such as neural networks (14, 38, 39) and statistical approaches such as joint models (14). Prediction models developed using landmark and joint models for single risk factors have been previously compared (40) but not in a setting using multivariate risk factors. Joint models are more computationally burdensome than landmark models, and further development is required before they are computationally feasible for application to large EHR data sets. However, landmark models can be developed using any standard statistical software with multivariate mixed-effects models and Cox regression. Analyses employing the landmark-age- and sex-specific multivariate mixed-effects models can be run in parallel, since the most computationally burdensome part is extracting the out-of-sample individual-specific random intercepts for estimation of the current risk factor values.

Certain limitations of our proposed method remain. First, our approach assumes a multivariate normal distribution for estimated current values of continuous and binary risk factors. Such an assumption is not uncommon in statistical methodology for epidemiology (e.g., in regression calibration (10) and multiple imputation (41)); however, it would be possible to replace it with a mixture of regression models with correlated latent variables (42). Second, the added distributional assumptions on the risk factors may limit transferability of the model to other populations and implicate recalibration methods for use of the model in other populations, especially in comparison with conventional CVD prediction models. Investigating the impact of model misspecification is on our future research agenda. Third, uncertainties in the estimated current risk factor values are not accounted for in the Cox model. However, our previous work suggested that such uncertainties are often negligible relative to the estimated standard errors of the β coefficients in the Cox model (10). Fourth, persons with more frequent EHRs are more likely to have health conditions or health anxiety. We attempted to account for this by adjusting the estimated current risk factor values by the annual rate of repeated measurements, although it may be plausible to additionally include this as a risk factor in the Cox model. Fifth, for our illustration, we assumed a lack of specific Read or drug codes to indicate no diagnosis or medication use, and information on cause of death was only available for 13% of participants who died, meaning CVD incidence was underestimated in this study. Sixth, we used the same definition of CVD events as used in CVD risk prediction models employed in practice, such as QRISK2, which includes “soft” outcomes such as angina. However, while angina can be a symptom of coronary heart disease, it is not a disease itself, and the appropriateness of including it in the outcome definition of CVD risk prediction models will depend on the clinical context. Finally, despite the use of contemporary data, CVD screening and treatment practices have changed over time and are not accounted for in the models. These limitations are unlikely to have affected our between-model comparisons.

The benefits of optimizing EHRs for disease risk screening and personalized health-care decisions are increasingly being recognized. There is a growing need for suitable statistical methods, data analytics, and machine learning approaches with which to address the computational and methodological challenges involved in the analysis of such “big data.” The framework presented in this paper provides a practical, transparent, and flexible solution for the development of dynamic risk prediction models for use on EHRs.

## Supplementary Material

Web Material

## Abbreviations

CI: confidence interval
CVD: cardiovascular disease
EHRs: electronic health records
HDL-C: high-density lipoprotein cholesterol
SBP: systolic blood pressure
