# Supplementary material for: Landmark Models for Optimizing the Use of Repeated Measurements of Risk Factors in Electronic Health Records to Predict Future Disease Risk
Source: Am J Epidemiol. 2018 Mar 23;187(7):1530–8. doi: 10.1093/aje/kwy018 (PMC6030927; doi:10.1093/aje/kwy018)
Supplement: Web Material [file kwy018paigewebmaterialfinal.pdf]

Landmark Models for Optimizing the Use of Repeated Measurements of Risk Factors in Electronic Health Records to Predict Future Disease Risk

Ellie Paige, Jessica Barrett, David Stevens, Ruth H. Keogh, Michael J. Sweeting, Irwin Nazareth,

Irene Petersen, and Angela M. Wood

WEB MATERIAL

Contents

Web Figure 1: Flowchart showing selection of patient records for analysis 2

Web Table 1: The RECORD checklist of items that should be reported in observational studies using routinely collected health data 3

Web Appendix: Detailed methods 9

Web Figure 2: Schematic showing the landmark age approach using 10-year future repeat measures of predictors 14

Web Table 2: Characteristics of people in the whole population and target population 15

Web Table 3: Results from the multivariate linear mixed-effects model of repeated data—main analysis.... 16

Web Table 4: Results from the multivariate linear mixed-effects model of repeated data—sensitivity analysis with adjustment for number of repeated measurements per year in the past five years 19

Web Table 5: Hazard ratios from the Cox proportional hazards models in the study sample 23

Web Table 6: Hazard ratios from the Cox proportional hazards models in the restricted sample 23

Web Figure 3: Calibration plots for each risk prediction model using the full data set 24

Web Figure 4: Overall and age-adjusted C-index across landmark ages—basic model 25

Web Figure 5: Distribution of absolute cardiovascular disease risk scores in the validation samples for each risk prediction model in the restricted data set 26

Web Figure 6: Calibration plots for each risk prediction model using the restricted data set 27

Web Figure 7: Calibration and risk discrimination statistics for each risk prediction model in the restricted sample* (*n* = 12,292) 28

Web Figure 8: Overall and age-adjusted C-index across landmark age models in the restricted sample 29

Web Table 7. Calibration and risk discrimination statistics for each risk prediction model in the study sample with statin users included (*n* = 36,297) 31

Web Table 8. Calibration and risk discrimination statistics for each risk prediction model in the restricted sample* with statin users included (*n* = 18,054) 31

Web Figure 1: Flowchart showing selection of patient records for analysis

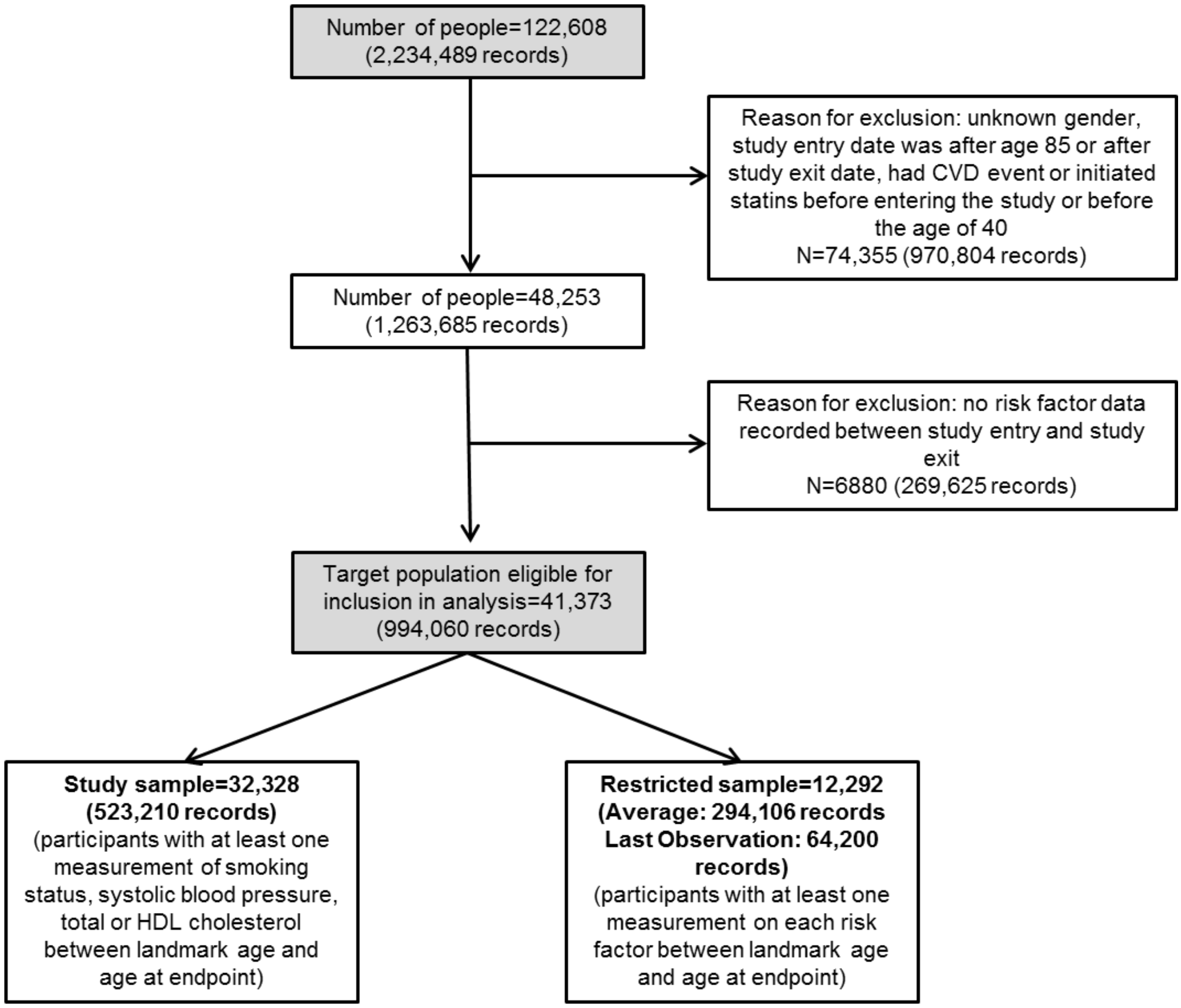

Web Table 1: The RECORD checklist of items that should be reported in observational studies using routinely collected health data

	Item No.	STROBE Items	Location in Manuscript Where Items Are Reported	RECORD Items	Location in Manuscript Where Items Are Reported
**Title and abstract**					
	1	(a) Indicate the study's design with a commonly used term in the title or the abstract (b) Provide in the abstract an informative and balanced summary of what was done and what was found	Title and abstract	RECORD 1.1: The type of data used should be specified in the title or abstract. When possible, the name of the databases used should be included.<br><br>RECORD 1.2: If applicable, the geographic region and timeframe within which the study took place should be reported in the title or abstract.<br><br>RECORD 1.3: If linkage between databases was conducted for the study, this should be clearly stated in the title or abstract.	Title<br><br>Abstract<br><br>N/A
**Introduction**					
Background rationale	2	Explain the scientific background and rationale for the investigation being reported	Introduction		
Objectives	3	State specific objectives, including any prespecified hypotheses	Introduction		
**Methods**					
Study Design	4	Present key elements of study design early in the paper	Methods, data source		
Setting	5	Describe the setting, locations, and relevant dates, including	Methods, data source		

		periods of recruitment, exposure, follow-up, and data collection			
Participants	6	*(a) Cohort study* - Give the eligibility criteria, and the sources and methods of selection of participants. Describe methods of follow-up<br>*Case-control study* - Give the eligibility criteria, and the sources and methods of case ascertainment and control selection. Give the rationale for the choice of cases and controls<br>*Cross-sectional study* - Give the eligibility criteria, and the sources and methods of selection of participants<br><br>*(b) Cohort study* - For matched studies, give matching criteria and number of exposed and unexposed<br>*Case-control study* - For matched studies, give matching criteria and the number of controls per case	Methods, study population and flowchart in Web Figure 1	RECORD 6.1: The methods of study population selection (such as codes or algorithms used to identify subjects) should be listed in detail. If this is not possible, an explanation should be provided.<br><br>RECORD 6.2: Any validation studies of the codes or algorithms used to select the population should be referenced. If validation was conducted for this study and not published elsewhere, detailed methods and results should be provided.<br><br>RECORD 6.3: If the study involved linkage of databases, consider use of a flow diagram or other graphical display to demonstrate the data linkage process, including the number of individuals with linked data at each stage.	Methods, study population and Web Figure 1<br><br>N/A<br><br>N/A
Variables	7	Clearly define all outcomes, exposures, predictors, potential confounders, and effect modifiers. Give diagnostic criteria, if applicable.	Methods, data source	RECORD 7.1: A complete list of codes and algorithms used to classify exposures, outcomes, confounders, and effect modifiers should be provided. If these cannot be reported, an explanation should be provided.	Code lists will be uploaded to ClinicalCodes.org following article publication
Data sources/ measurement	8	For each variable of interest, give sources of data and details of	Methods, study population		

		methods of assessment (measurement). Describe comparability of assessment methods if there is more than one group			
Bias	9	Describe any efforts to address potential sources of bias	Methods, statistical analysis		
Study size	10	Explain how the study size was arrived at	Web Figure 1		
Quantitative variables	11	Explain how quantitative variables were handled in the analyses. If applicable, describe which groupings were chosen, and why	Methods		
Statistical methods	12	(a) Describe all statistical methods, including those used to control for confounding<br>(b) Describe any methods used to examine subgroups and interactions<br>(c) Explain how missing data were addressed<br>(d) *Cohort study* - If applicable, explain how loss to follow-up was addressed<br>*Case-control study* - If applicable, explain how matching of cases and controls was addressed<br>*Cross-sectional study* - If applicable, describe analytical methods taking account of sampling strategy	Methods, statistical analysis and Web Appendix		

		(e) Describe any sensitivity analyses			
Data access and cleaning methods		..		RECORD 12.1: Authors should describe the extent to which the investigators had access to the database population used to create the study population.<br><br>RECORD 12.2: Authors should provide information on the data cleaning methods used in the study.	Methods, study population<br><br>Methods, study population
Linkage		..		RECORD 12.3: State whether the study included person-level, institutional-level, or other data linkage across two or more databases. The methods of linkage and methods of linkage quality evaluation should be provided.	N/A
**Results**					
Participants	13	(a) Report the numbers of individuals at each stage of the study (*e.g.*, numbers potentially eligible, examined for eligibility, confirmed eligible, included in the study, completing follow-up, and analyzed)<br>(b) Give reasons for non-participation at each stage.<br>(c) Consider use of a flow diagram	Web Figure 1	RECORD 13.1: Describe in detail the selection of the persons included in the study (*i.e.*, study population selection) including filtering based on data quality, data availability and linkage. The selection of included persons can be described in the text and/or by means of the study flow diagram.	Web Figure 1
Descriptive data	14	(a) Give characteristics of study participants (*e.g.*, demographic, clinical, social) and information on exposures and potential confounders	Results and Table 1		

		(b) Indicate the number of participants with missing data for each variable of interest<br>(c) *Cohort study* - summarize follow-up time (*e.g.*, average and total amount)	Results and Web Figure 1<br><br><br>Pg. 10		
Outcome data	15	*Cohort study* - Report numbers of outcome events or summary measures over time<br>*Case-control study* - Report numbers in each exposure category, or summary measures of exposure<br>*Cross-sectional study* - Report numbers of outcome events or summary measures	Results		
Main results	16	(a) Give unadjusted estimates and, if applicable, confounder-adjusted estimates and their precision (e.g., 95% confidence interval). Make clear which confounders were adjusted for and why they were included<br>(b) Report category boundaries when continuous variables were categorized<br>(c) If relevant, consider translating estimates of relative risk into absolute risk for a meaningful time period	Results and Figures 2-3		
Other analyses	17	Report other analyses done—e.g., analyses of subgroups and interactions, and sensitivity analyses	Results, sensitivity analyses		

**Discussion**					
Key results	18	Summarize key results with reference to study objectives	Results		
Limitations	19	Discuss limitations of the study, taking into account sources of potential bias or imprecision. Discuss both direction and magnitude of any potential bias	Discussion	RECORD 19.1: Discuss the implications of using data that were not created or collected to answer the specific research question(s). Include discussion of misclassification bias, unmeasured confounding, missing data, and changing eligibility over time, as they pertain to the study being reported.	Discussion
Interpretation	20	Give a cautious overall interpretation of results considering objectives, limitations, multiplicity of analyses, results from similar studies, and other relevant evidence	Discussion		
Generalizability	21	Discuss the generalizability (external validity) of the study results	Discussion		
**Other Information**					
Funding	22	Give the source of funding and the role of the funders for the present study and, if applicable, for the original study on which the present article is based	Funding statement published		
Accessibility of protocol, raw data, and programming code		..		RECORD 22.1: Authors should provide information on how to access any supplemental information such as the study protocol, raw data, or programming code.	N/A

Web Appendix: Detailed methods

1. Multivariate mixed model and landmark age approach

Our aim was to build a predictive model from which to estimate 10-year CVD risk for CVD-event free individuals and those who have not yet initiated statins, using their historical electronic health records of key CVD risk factors. Importantly, the predictive model needs to handle the incomplete repeat measures structure of the data and also have the flexibility to account for number of GP visits as a proxy for illness severity or health anxiety among healthy people.

We used a sliding landmark age approach.[1] In this approach a prediction model was derived at 5-yearly intervals (which we call landmark ages and denote as L_a) and represents a series of prediction models for 40-, 45-, …, 85-year-olds. Only participants with no CVD diagnoses and/or statin prescription prior to that landmark age were included in the derivation of the models. From each landmark age, participants were followed up for 10 years until their first (i.e. 'incident') newly recorded CVD event, transfer out of the practice, their date of death, or 31 December 2011 whichever came first.

We selected the key CVD risk factors as those used in the validated ACC/AHA Pooled Cohort Equations[2]: age, sex, diabetes status, smoking status, systolic blood pressure (adjusted for blood pressure-lowering medication prescriptions), total cholesterol and HDL cholesterol. Values of systolic blood pressure, total cholesterol and HDL cholesterol were standardized by centering on sex-specific means and dividing by the standard deviation. Age and sex were known for all participants included in the models. Values for diabetes mellitus status and history of blood pressure-lowering medication prescription were set to zero until health records indicated otherwise (i.e. for diabetes mellitus: at least one diabetes diagnostic code [Read code or diabetes test] plus either an additional diagnostic code or one or more prescriptions of a diabetes drug[3]; first prescription of a blood-pressure lowering medication) from which time the values were set to one. Repeat measures made before landmark age L_a for smoking status, systolic blood pressure, total cholesterol and HDL cholesterol were first summarized using a multivariate mixed model[4] and entered the prediction model as single summary measures as described below.

The landmark age approach comprises of two stages:

Stage 1: Multivariate mixed model

We let $smoking\_status_{ij}$, $systolic\_blood\_pressure_{ij}$, $total\_cholesterol_{ij}$, $high\_density\_lipoprotein_{ij}$, BPM_{ij} and age_{ij} denote the repeat measures of smoking status, systolic blood pressure, total cholesterol, HDL cholesterol, history of blood pressure-lowering medication prescription and age for individual i recorded at measurement j. For males and females separately, for each landmark age L_a = 40, 45, 50, …,85 and repeat measures recorded before L_a, we fit a multivariate mixed model with a correlated covariance structure:

$$\begin{aligned} smoking\_status_{ij} &= a_1 + b_1 \times age_{ij} + u_{1i} + e_{1ij} \\ systolic\_blood\_pressure_{ij} &= a_2 + b_2 \times age_{ij} + c \times BPM_{ij} + u_{2i} + e_{2ij} \\ total\_cholesterol_{ij} &= a_3 + b_3 \times age_{ij} + u_{3i} + e_{3ij} \\ high\_density\_lipoprotein_{ij} &= a_4 + b_4 \times age_{ij} + u_{4i} + e_{4ij} \end{aligned} \qquad for\ j \le L_a$$

$$\text{where} \begin{bmatrix} u_{1i} \\ u_{2i} \\ u_{3i} \\ u_{4i} \end{bmatrix} \sim \text{multivariate normal} \left(\begin{bmatrix} 0 \\ 0 \\ 0 \\ 0 \end{bmatrix}, \begin{bmatrix} \sigma_1^2 & \sigma_{12} & \sigma_{13} & \sigma_{14} \\ \sigma_{12} & \sigma_2^2 & \sigma_{23} & \sigma_{24} \\ \sigma_{13} & \sigma_{23} & \sigma_3^2 & \sigma_{34} \\ \sigma_{14} & \sigma_{24} & \sigma_{34} & \sigma_4^2 \end{bmatrix} \right),$$

$$\text{and} \begin{bmatrix} e_{1ij} \\ e_{2ij} \\ e_{3ij} \\ e_{4ij} \end{bmatrix} \sim \text{multivariate normal} \left(\begin{bmatrix} 0 \\ 0 \\ 0 \\ 0 \end{bmatrix}, \begin{bmatrix} \sigma_{e1}^2 & 0 & 0 & 0 \\ 0 & \sigma_{e2}^2 & 0 & 0 \\ 0 & 0 & \sigma_{e3}^2 & 0 \\ 0 & 0 & 0 & \sigma_{e4}^2 \end{bmatrix} \right).$$

Here a_1, a_2, a_3 and a_4 represent fixed intercepts for each risk factor, b_1, b_2, b_3 and b_4 represent fixed slope for each risk factor and c represents an adjustment factor in systolic blood pressure levels for those with a history of blood pressure-lowering medication prescription.

Terms u_{1i}, u_{2i}, u_{3i} and u_{4i} represent random intercepts for each risk factor and are correlated between risk factors. These random intercepts are interpreted as the difference in the average historical level of the predictor for this individual above the population average historical level.

Finally, e_{1ij}, e_{2ij}, e_{3ij} and e_{4ij} represent uncorrelated residual errors for each risk factor.

This model allows incomplete records of the risk factors, and includes all individuals with at least one measurement from at least one risk factor. The correlation structure between the risk factors is estimated from individuals with observed data on more than one risk factor. Thus, the model assumes that, for each landmark age, risk factor values from individuals with incomplete data are from the same multivariate normal distribution for risk factor values for individuals with observed data (that is, assuming "missing at random").

Our model assumes that all risk factors jointly follow a multivariate normal distribution, which is plausible for systolic blood pressure, total cholesterol, HDL cholesterol but less plausible for smoking status which is defined as a binary variable. However, inference based from the multivariate normal distribution may often be reasonable even if the multivariate normality does not hold, especially in the context of imputation of missing data[5] and regression calibration.[6, 7]

Best linear unbiased predictors (BLUPS)[8] can be estimated for each risk factor from the random intercepts $\widehat{u_{1i}}, \widehat{u_{2i}}, \widehat{u_{3i}}$ and $\widehat{u_{4i}}$ using observed data for $j \leq L_a$.[9] The BLUPs are estimated as the mean of the empirical Bayes posterior distribution of the random intercepts conditional on observed risk factor measurements. Using the properties of multivariate normal distributions, this is also a multivariate normal distribution, and an exact formula for the mean can be calculated.[10] Specifically, for individual i

$$\begin{bmatrix} \hat{u}_{1i} \\ \hat{u}_{2i} \\ \hat{u}_{3i} \\ \hat{u}_{4i} \end{bmatrix} = GZ^T(ZGZ^T + \Sigma)^{-1}(Y - X\beta)$$

Here Y is the vector of risk factor observations, G is the covariance matrix of the random effects $= \begin{bmatrix} \sigma_1^2 & \sigma_{12} & \sigma_{13} & \sigma_{14} \\ \sigma_{12} & \sigma_2^2 & \sigma_{23} & \sigma_{24} \\ \sigma_{13} & \sigma_{23} & \sigma_3^2 & \sigma_{34} \\ \sigma_{14} & \sigma_{24} & \sigma_{34} & \sigma_4^2 \end{bmatrix}$, Z is the design matrix which selects the corresponding random effect for each risk factor, Z^T is the matrix transpose of Z and Σ is a diagonal matrix containing the corresponding residual variance for each risk factor. Importantly, due to the correlations structure between the random intercepts, BLUPS can be estimated for all individuals with at least one repeat measure for at least one risk factor.

Stage 2: Cox proportional hazards "Super-landmark model"

A super-landmark model is a version of landmarking in which the data sets contributing to the landmark models across all landmark ages are stacked and a single time-to-event model is fitted to the stacked data set.[1] An advantage is that a single set of regression coefficients are estimated, aiding interpretation. A Cox proportional hazards super-landmark model was fitted to the time-to-event data. First, landmark age data sets were constructed comprising of participants with no CVD diagnoses and/or statin prescription prior to that landmark age and included the following variables: (i) the landmark-age-dependent outcome time-to-event and censoring indicator (ii) sex and (iii) landmark-age-dependent risk factors denoted together as $X(L_a)$ which includes BLUPS, estimated in stage 1, for smoking status, systolic blood pressure, total cholesterol and HDL cholesterol, and the most recent observed records for diabetes status and history of blood pressure-lowering medication prescriptions.. Secondly, the landmark age data sets were stacked into a single data set, to which the Cox proportional hazards super-landmark model, stratified by sex s, was fitted:

$$h_s(t|X(L_a), L_a) \; = \; h_{0s}(t)\exp({\beta_0}^T X(L_a) + \theta_1 L_a + \theta_2 {L_a}^2)$$

This model assumes that baseline hazards at different landmark ages differ only by an adjustment for landmark age and landmark age squared. The effect of each covariate included in the model is assumed to be the same at each landmark age. More flexible models also included a landmark-age interaction with each covariate in $X(L_a)$. The model is estimated using robust standard errors to account for the same individuals being used at multiple landmark ages.

Predicted 10-year CVD risk is estimated for participants at each landmark age from the equation:

$$1 - P\big(T > L_a + 10\big|T > L_a, X(L_a)\big) \; = \; 1 - \widehat{S_{0s}}(L_a + 10|\, L_a\,)^{\exp(\widehat{\beta_0}^T X(L_a) + \widehat{\theta_1} L_a + \widehat{\theta_2} {L_a}^2)}$$

where $\widehat{S_{0s}}(L_a + 10|\, L_a\,)$ represents the sex-stratified 10-year baseline survival from landmark age L_a.

2. C-index

The C-index estimates the probability of concordance of predicted risk and observed order of events for a randomly selected pair of participants, and can be interpreted as the probability that the order of events is correctly predicted in a randomly selected pair of individuals. It is calculated by making all possible pairwise comparisons between participants (censoring permitting) and classing each pair according to whether their predicted and observed order of event matches.

This is simple to apply in stacked landmark data sets, however, we need to ensure all pairwise comparisons are made between two different participants and not within the same participant.

3. Ten-fold cross validation

We used ten-fold cross-validation with the data split by general practice to allow all available data to be analyzed while accounting for over-optimism in the performance of the model. In summary, the multivariate mixed model and the Cox proportional hazards were derived using participants from nine general practices. The estimated parameters were then used to predict landmark-age specific 10-year CVD risk for participants from the excluded tenth general practice, requiring out-of-sample estimation of the BLUPS from the multivariate mixed model at each landmark age.[10] This process was repeated 10 times, excluding a different general practice each time. The predicted landmark-age specific 10-year CVD risk for participants from all 10 general practices were then combined and used for the assessment of calibration and discrimination.

4. **Sensitivity analyses**

   a) *using repeat measurements from the past ten years before each landmark age*

   We performed a sensitivity analysis whereby the multivariate mixed model was fitted to participants' repeat measures recorded in the past 10 years before the landmark age, i.e.: $L_a - 10 \leq j \leq L_a$.

   b) *adjusting the multivariate mixed-effects model for the total number of repeated measurements before the landmark age*

   Additional covariates representing the number of repeat measurements made per year, estimated from the previous 5 years, were included in the multivariate mixed model. This was to account for sicker or more health conscious individuals having more repeats due to more frequent GP visits.

   c) *incorporating 10-year future repeat measures of predictors in the derivation of the multivariate mixed-effects model*

   We performed a sensitivity analysis whereby the multivariate mixed model was derived using participants' repeat measures recorded in the past and the future, i.e.: $j \leq L_a + 10$. This was in order to attempt to obtain more relevant BLUPS which closely represent the usual level of the risk predictors over the life course of each participant. Note, that for validation the repeat measures were intuitively restricted to $j \leq L_a$.

References

1. van Houwelingen HC, Putter H. *Dynamic prediction in clinical survival analysis*. Florida, US: CRC Press, Taylor & Francis Group; 2012.
2. Muntner P, Colantonio LD, Cushman M, et al. Validation of the atherosclerotic cardiovascular disease Pooled Cohort risk equations. *Jama* 2014; **311**: 1406-15.
3. Sharma M, Petersen I, Nazareth I, Coton SJ. An algorithm for identification and classification of individuals with type 1 and type 2 diabetes mellitus in a large primary care database. *Clinical Epidemiology* 2016; **8**: 373-80.
4. Verbeke G, Fieuws S, Molenberghs G, Davidian M. The analysis of multivariate longitudinal data: a review. *Statistical methods in medical research* 2014; **23**: 42-59.
5. Schafer JL. *Analysis of incomplete multivariate data*: CRC press; 1997.
6. Fibrinogen Studies C. Correcting for multivariate measurement error by regression calibration in meta-analyses of epidemiological studies. *Statistics in medicine* 2009; **28**: 1067-92.
7. White I, Frost C, Tokunaga S. Correcting for measurement error in binary and continuous variables using replicates. *Stat Med* 2001; **20**: 3441-57.

8. Goldberger AS. Best linear unbiased prediction in the generalized linear regression model. *Journal of the American Statistical Association* 1962; **57**: 369-75.
9. Goldstein H. *Multilevel statistical models*: John Wiley & Sons; 2011.
10. Diggle P, Heagerty P, Liang KY, Zeger SL. *Analysis of longitudinal data*: Oxford University Press; 2002.

Web Figure 2: Schematic showing the landmark age approach using 10-year future repeat measures of predictors

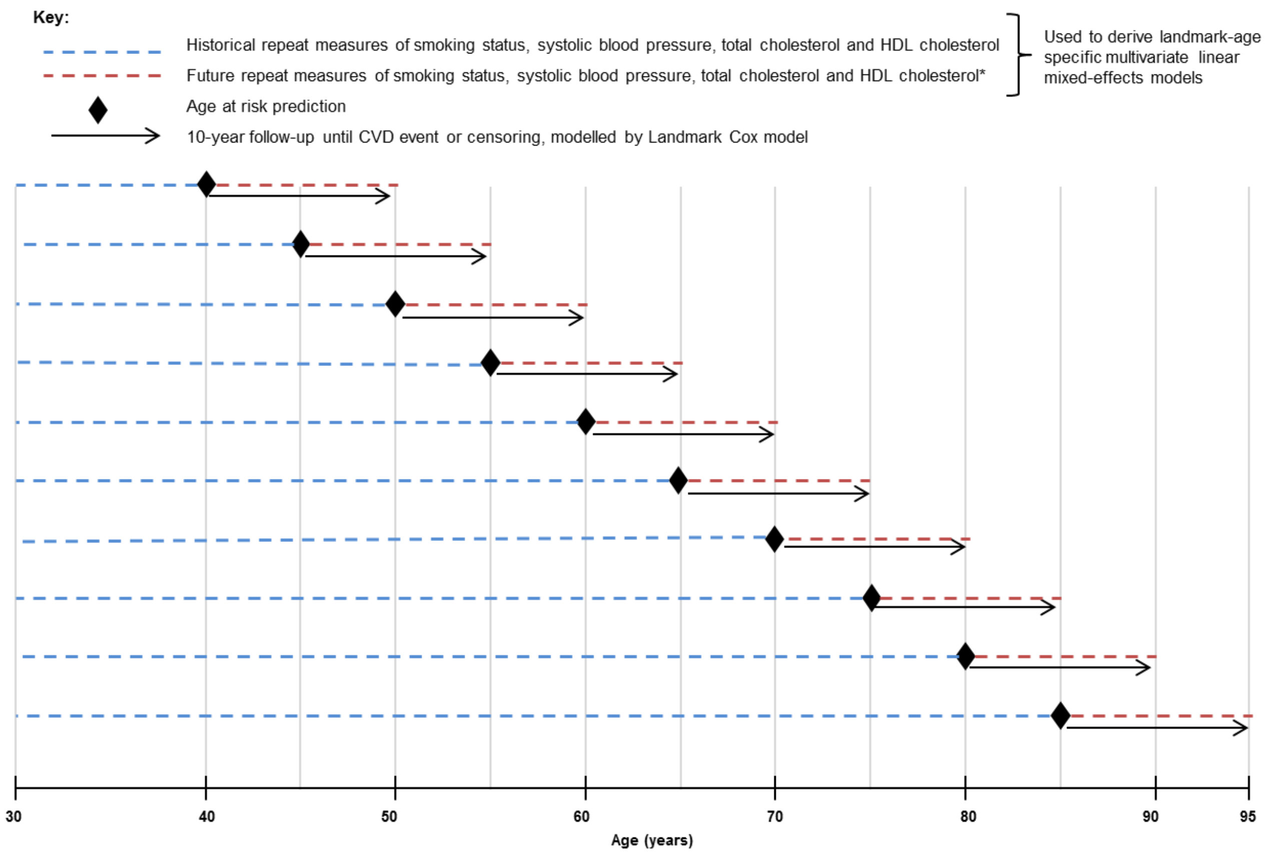

*Future risk factors values were only used for model derivation included in the model if they were recorded before first CVD event, censoring, or within 10 years of landmark age.

Web Table 2: Characteristics of people in the whole population and target population

	Baseline Characteristics	
	**Whole Population (*n* = 122,608)**	**Target Population (*n* = 41,373)**
**Mean age at study entry, years (SD)**	45.4 (18.0)	48.8 (13.8)
**Males, no. (%)**	56,186 (47)	17,194 (47)
**History of diabetes, no. (%)***	15,272 (13)	4,606 (13)
**Blood pressure-lowering medication prescriptions, no. (%)***	23,601 (20)	11,753 (32)
**Statin prescriptions, no. (%)***	17,161 (14)	7,718 (21)
**Current smokers, no. (%)***	-	10,615 (31)
**Systolic blood pressure, mean mm Hg (SD)^**	-	135.7 (21.5)
**Total cholesterol, mean mmol/liter (SD)^**	-	5.5 (1.1)
**HDL-C, mean mmol/liter (SD)^**	-	1.4 (0.4)

HDL-C = high-density lipoprotein cholesterol; SD = standard deviation.

*Number and % calculated across follow-up period (i.e. a diagnosis of diabetes at any point during follow-up is counted as a history of diabetes for that individual).

^based on first measurements after study entry.

Web Table 3: Results from the multivariate linear mixed-effects model of repeated data—main analysis

	Coefficient (95% CI) Women	Coefficient (95% CI) Men
**Landmark age 40**		
**Smoking**		
Intercept	0.25 (0.24, 0.27)	0.33 (0.31, 0.35)
Age	0.010 (0.008, 0.012)	0.012 (0.009, 0.015)
Random effects variance	0.36 (0.34, 0.38)	0.48 (0.45, 0.52)
**Systolic blood pressure**		
Intercept	-0.57 (-0.59, -0.55)	-0.35 (-0.38, -0.32)
Age	-0.020 (-0.022, -0.017)	-0.001 (-0.007, 0.005)
Treatment for blood pressure	-0.10 (-0.14, -0.05)	-0.22 (-0.28, -0.17)
Random effects variance	0.24 (0.22, 0.25)	0.29 (0.27, 0.31)
**Total cholesterol**		
Intercept	-0.21 (-0.26, -0.17)	0.38 (0.33, 0.44)
Age	-0.014 (-0.023, -0.006)	-0.021 (-0.032, 0.010)
Random effects variance	0.54 (0.50, 0.59)	0.75 (0.68, 0.83)
**HDL cholesterol**		
Intercept	-0.18 (-0.25, -0.11)	-0.14 (-0.21, -0.08)
Age	0.004 (-0.011, 0.019)	0.023 (0.007, 0.040)
Random effects variance	0.96 (0.87, 1.06)	0.82 (0.73, 0.92)
**Landmark age 45**		
**Smoking**		
Intercept	0.27 (0.25, 0.28)	0.30 (0.28, 0.32)
Age	0.006 (0.004, 0.008)	0.011 (0.008, 0.013)
Random effects variance	0.43 (0.41, 0.46)	0.49 (0.46, 0.52)
**Systolic blood pressure**		
Intercept	-0.36 (-0.38, -0.34)	-0.29 (-0.32, -0.27)
Age	-0.026 (-0.028, -0.024)	-0.003 (-0.008, 0.001)
Treatment for blood pressure	-0.15 (-0.18, -0.12)	-0.22 (-0.27, -0.18)
Random effects variance	0.29 (0.28, 0.31)	0.31 (0.29, 0.33)
**Total cholesterol**		
Intercept	-0.06 (-0.10, -0.03)	0.42 (0.38, 0.47)
Age	-0.025 (-0.032, -0.019)	-0.011 (-0.019, -0.002)
Random effects variance	0.65 (0.60, 0.70)	0.73 (0.67, 0.79)
**HDL cholesterol**		
Intercept	-0.05 (-0.10, 0.00)	-0.05 (-0.10, 0.00)
Age	-0.004 (-0.015, 0.006)	0.013 (0.001, 0.025)
Random effects variance	1.12 (1.04, 1.22)	0.84 (0.76, 0.92)
**Landmark age 50**		
**Smoking**		
Intercept	0.25 (0.24, 0.27)	0.27 (0.25, 0.29)
Age	0.006 (0.005, 0.008)	0.010 (0.008, 0.012)
Random effects variance	0.53 (0.50, 0.56)	0.52)0.49, 0.55)
**Systolic blood pressure**		
Intercept	-0.13 (-0.15, -0.10)	-0.20 (-0.22, -0.17)
Age	-0.029 (-0.032, -0.027)	-0.009 (-0.013, -0.005)
Treatment for blood pressure	-0.26 (-0.15, -0.10)	-0.31 (-0.35, -0.28)
Random effects variance	0.36 (0.34, 0.38)	0.34 (0.32, 0.36)
**Total cholesterol**		
Intercept	0.14 (0.11, 0.18)	0.46 (0.42, 0.50)
Age	-0.036 (-0.041, -0.031)	-0.002 (-0.008, 0.005)
Random effects variance	0.73 (0.68, 0.79)	0.73 (0.67, 0.79)
**HDL cholesterol**		
Intercept	0.10 (0.04, 0.15)	0.03 (-0.02, 0.08)
Age	-0.017 (-0.026, -0.008)	0.001 (-0.001, 0.009)
Random effects variance	1.26 (1.16, 1.37)	0.95 (0.88, 1.04)
**Landmark age 55**		
**Smoking**		
Intercept	0.22 (0.21, 0.24)	0.25 (0.23, 0.27)
Age	0.010 (0.009, 0.012)	0.010 (0.007, 0.012)
Random effects variance	0.58 (0.54, 0.61)	0.50 (0.47, 0.54)
**Systolic blood pressure**		
Intercept	0.10 (0.07, 0.13)	-0.09 (-0.12, -0.06)

	Coefficient (95% CI) Women	Coefficient (95% CI) Men
Age	-0.025 (-0.028, -0.022)	-0.006 (-0.010, -0.002)
Treatment for blood pressure	-0.27 (-0.30, -0.24)	-0.30 (-0.33, -0.26)
Random effects variance	0.42 (0.40, 0.45)	0.35 (0.33, 0.38)
**Total cholesterol**		
Intercept	0.41 (0.437, 0.44)	0.44 (0.40, 0.48)
Age	-0.052 (-0.058, -0.047)	0.002 (-0.003, 0.008)
Random effects variance	0.81 (0.76, 0.87)	0.75 (0.69, 0.82)
**HDL cholesterol**		
Intercept	0.18 (0.12, 0.24)	0.08 (0.03, 0.14)
Age	-0.010 (-0.019, -0.001)	-0.001 (-0.009, 0.007)
Random effects variance	1.38 (1.27, 1.50)	0.88 (0.80, 0.97)
**Landmark age 60**		
**Smoking**		
Intercept	0.20 (0.18, 0.21)	0.24 (0.22, 0.26)
Age	0.010 (0.008, 0.012)	0.010 (0.008, 0.012)
Random effects variance	0.55 (0.52, 0.58)	0.57 (0.53, 0.61)
**Systolic blood pressure**		
Intercept	0.25 (0.22, 0.27)	0.09 (0.06, 0.13)
Age	-0.016 (-0.019, -0.013)	-0.009 (-0.013, -0.005)
Treatment for blood pressure	-0.28 (-0.31, -0.26)	-0.39 (-0.42, -0.35)
Random effects variance	0.43 (0.40, 0.45)	0.42 (0.39, 0.45)
**Total cholesterol**		
Intercept	0.46 (0.43, 0.50)	0.40 (0.35, 0.44)
Age	-0.036 (-0.041, -0.031)	0.007 (0.002, 0.013)
Random effects variance	0.82 (0.76, 0.88)	0.84 (0.77, 0.91)
**HDL cholesterol**		
Intercept	0.20 (0.14, 0.26)	0.13 (0.07, 0.18)
Age	-0.000 (-0.008, 0.012)	-0.002 (-0.011, 0.001)
Random effects variance	1.41 (1.29, 1.54)	0.99 (0.89, 1.09)
**Landmark age 65**		
**Smoking**		
Intercept	0.16 (0.14, 0.17)	0.20 (0.18, 0.22)
Age	0.013 (0.011, 0.014)	0.012 (0.010, 0.015)
Random effects variance	0.52 (0.48, 0.56)	0.51 (0.47, 0.56)
**Systolic blood pressure**		
Intercept	0.37 (0.34, 0.40)	0.14 (0.10, 0.17)
Age	-0.008 (-0.011, -0.005)	-0.001 (-0.005, 0.003)
Treatment for blood pressure	-0.29 (-0.32, -0.26)	-0.33 (-0.36, -0.29)
Random effects variance	0.40 (0.38, 0.43)	0.37 (0.35, 0.41)
**Total cholesterol**		
Intercept	0.45 (0.41, 0.49)	0.26 (0.22, 0.30)
Age	-0.018 (-0.023, -0.013)	0.008 (0.003, 0.013)
Random effects variance	0.90 (0.84, 0.98)	0.81 (0.74, 0.89)
**HDL cholesterol**		
Intercept	0.21 (0.15, 0.27)	0.21 (0.15, 0.28)
Age	0.007 (-0.001, 0.014)	-0.003 (-0.011, 0.005)
Random effects variance	1.27 (1.16, 1.39)	1.05 (0.94, 1.17)
**Landmark age 70**		
**Smoking**		
Intercept	0.14 (0.12, 0.16)	0.17 (0.14, 0.19)
Age	0.012 (0.010, 0.014)	0.014 (0.012, 0.017)
Random effects variance	0.50 (0.46, 0.54)	0.51 (0.46, 0.56)
**Systolic blood pressure**		
Intercept	0.50 (0.47, 0.54)	0.24 (0.20, 0.29)
Age	0.003 (-0.003, 0.007)	0.010 (0.005, 0.015)
Treatment for blood pressure	-0.27 (-0.30, -0.24)	-0.35 (-0.39, -0.31)
Random effects variance	0.37 (0.34, 0.40)	0.39 (0.36, 0.43)
**Total cholesterol**		
Intercept	0.39 (0.34, 0.44)	0.20 (0.15, 0.26)
Age	-0.002 (-0.007, 0.003)	0.016 (0.010, 0.022)
Random effects variance	0.84 (0.77, 0.93)	0.84 (0.76, 0.94)
**HDL cholesterol**		
Intercept	0.22 (0.15, 0.29)	0.22 (0.14, 0.30)
Age	0.005 (-0.004, 0.013)	0.007 (-0.004, 0.018)

	Coefficient (95% CI) Women	Coefficient (95% CI) Men
Random effects variance	1.28 (1.15, 1.43)	1.15 (1.01, 1.31)
**Landmark age 75**		
**Smoking**		
Intercept	0.12 (0.10, 0.14)	0.12 (0.08, 0.13)
Age	0.010 (0.008, 0.012)	0.014 (0.011, 0.017)
Random effects variance	0.53 (0.48, 0.58)	0.51 (0.45, 0.57)
**Systolic blood pressure**		
Intercept	0.69 (0.65, 0.73)	0.32 (0.27, 0.37)
Age	0.011 (0.007, 0.016)	0.020 (0.015, 0.025)
Treatment for blood pressure	-0.32 (-0.36, -0.28)	-0.34 (-0.39, -0.29)
Random effects variance	0.42 (0.38, 0.46)	0.44 (0.39, 0.49)
**Total cholesterol**		
Intercept	0.34 (0.29, 0.40)	0.11 (0.06, 0.17)
Age	0.006 (0.011, 0.012)	0.017 (0.011, 0.024)
Random effects variance	0.82 (0.73, 0.91)	0.80 (0.70, 0.92)
**HDL cholesterol**		
Intercept	0.30 (0.22, 0.39)	0.22 (0.13, 0.32)
Age	-0.013 (0.024, -0.003)	0.014 (0.003, 0.025)
Random effects variance	1.20 (1.05, 1.38)	1.24 (1.07, 1.45)
**Landmark age 80**		
**Smoking**		
Intercept	0.09 (0.07, 0.11)	0.12 (0.09, 0.15)
Age	0.010 (0.008, 0.012)	0.006 (0.003, 0.009)
Random effects variance	0.53 (0.48, 0.59)	0.47 (0.41, 0.54)
**Systolic blood pressure**		
Intercept	0.81 (0.76, 0.86)	0.40 (0.34, 0.45)
Age	0.023 (0.018, 0.027)	0.023 (0.017, 0.029)
Treatment for blood pressure	-0.35 (-0.39, -0.31)	-0.35 (-0.40, -0.30)
Random effects variance	0.45 (0.41, 0.50)	0.38 (0.34, 0.43)
**Total cholesterol**		
Intercept	0.32 (0.25, 0.38)	0.05 (-0.01, 0.12)
Age	0.024 (0.018, 0.031)	0.024 (0.017, 0.031)
Random effects variance	1.01 (0.90, 1.14)	0.66 (0.56, 0.78)
**HDL cholesterol**		
Intercept	0.27 (0.17, 0.37)	0.32 (0.19, 0.45)
Age	0.001 (-0.010, 0.013)	0.001 (-0.013, 0.014)
Random effects variance	1.37 (1.16, 1.61)	1.21 (0.99, 1.47)
**Landmark age 85**		
**Smoking**		
Intercept	0.06 (0.04, 0.08)	0.09 (0.06, 0.12)
Age	0.009 (0.006, 0.011)	0.005 (0.002, 0.007)
Random effects variance	0.45 (0.40, 0.51)	0.55 (0.46, 0.65)
**Systolic blood pressure**		
Intercept	0.76 (0.70 0.82)	0.31 (0.23, 0.40)
Age	0.033 (0.028, 0.038)	0.047 (0.040, 0.054)
Treatment for blood pressure	-0.30 (-0.35, -0.26)	-0.40 (-0.47, -0.33)
Random effects variance	0.39 (0.35, 0.44)	0.47 (0.40, 0.55)
**Total cholesterol**		
Intercept	0.28 (0.20, 0.36)	-0.04 (-0.12, 0.05)
Age	0.027 (0.018, 0.035)	0.031 (0.021, 0.040)
Random effects variance	0.85 (0.73, 0.98)	0.70 (0.57, 0.87)
**HDL cholesterol**		
Intercept	0.27 (0.14, 0.40)	0.11 (-0.01, 0.23)
Age	-0.002 (-0.017, 0.013)	0.009 (-0.005, 0.024)
Random effects variance	1.50 (1.25, 0.79)	0.79 (0.61, 1.02)

HDL = high-density lipoprotein.

Web Table 4: Results from the multivariate linear mixed-effects model of repeated data—sensitivity analysis with adjustment for number of repeated measurements per year in the past five years

	Coefficient (95% CI) Women	Coefficient (95% CI) Men
**Landmark age 40**		
**Smoking**		
Intercept	0.28 (0.26, 0.30)	0.33 (0.31, 0.36)
Age	0.009 (0.005, 0.013)	0.018 (0.011, 0.025)
Number of repeats	-0.002 (-0.009, 0.004)	-0.000 (-0.010, 0.009)
Random effects variance	0.39 (0.36, 0.41)	0.49 (0.46, 0.54)
**Systolic blood pressure**		
Intercept	-0.59 (-0.62, -0.57)	-0.47 (-0.51, -0.42)
Age	-0.023 (-0.029, -0.018)	0.013 (0.001, 0.024)
Treatment for blood pressure	-0.11 (-0.16, -0.05)	-0.18 (-0.24, -0.12)
Number of repeats	0.017 (0.011, 0.023)	0.057 (0.045, 0.070)
Random effects variance	0.25 (0.24, 0.27)	0.29 (0.27, 0.32)
**Total cholesterol**		
Intercept	-0.22 (-0.28, -0.15)	0.32 (0.24, 0.40)
Age	-0.015 (-0.031, 0.001)	0.000 (-0.022, 0.023)
Number of repeats	0.004 (-0.010, 0.018)	0.018 (-0.003, 0.038)
Random effects variance	0.56 (0.51, 0.62)	0.79 (0.71, 0.88)
**HDL cholesterol**		
Intercept	-0.13 (-0.23, 0.03)	-0.05 (-0.15, 0.05)
Age	-0.007 (-0.032, 0.018)	-0.012 (-0.040, 0.016)
Number of repeats	-0.018 (-0.040, 0.004)	-0.024 (-0.051, 0.003)
Random effects variance	0.96 (0.86, 1.07)	0.83 (0.73, 0.95)
**Landmark age 45**		
**Smoking**		
Intercept	0.26 (0.24, 0.28)	0.32 (0.29, 0.34)
Age	0.008 (0.004, 0.012)	0.013 (0.008, 0.019)
Number of repeats	0.009 (0.003, 0.015)	-0.003 (-0.011, 0.006)
Random effects variance	0.46 (0.43, 0.49)	0.50 (0.47, 0.54)
**Systolic blood pressure**		
Intercept	-0.44 (-0.47, -0.42)	-0.43 (-0.47, -0.40)
Age	-0.028 (-0.034, -0.021)	0.021 (0.011, 0.031)
Treatment for blood pressure	-0.13 (-0.17, -0.09)	-0.19 (-0.24, -0.14)
Number of repeats	0.047 (0.040, 0.055)	0.056 (0.045, 0.067)
Random effects variance	0.30 (0.29, 0.32)	0.31 (0.28, 0.33)
**Total cholesterol**		
Intercept	-0.10 (-0.16, -0.05)	0.37 (0.30, 0.44)
Age	-0.019 (-0.033, -0.005)	-0.005 (-0.023, 0.013)
Number of repeats	0.015 (0.004, 0.027)	0.023 (0.005, 0.041)
Random effects variance	0.67 (0.62, 0.73)	0.73 (0.66, 0.80)
**HDL cholesterol**		
Intercept	-0.04 (-0.11, 0.04)	0.01 (-0.06, 0.09)
Age	0.001 (-0.017, 0.020)	0.010 (-0.011, 0.031)
Number of repeats	-0.016 (-0.033, 0.001)	-0.003 (-0.011, 0.006)
Random effects variance	1.18 (1.09, 1.28)	0.86 (0.78, 0.95)
**Landmark age 50**		
**Smoking**		
Intercept	0.24 (0.22, 0.26)	0.30 (0.27, 0.32)
Age	0.013 (0.009, 0.017)	0.011 (0.006, 0.017)
Number of repeats	0.009 (0.003, 0.015)	-0.005 (-0.012, 0.003)
Random effects variance	0.54 (0.51, 0.58)	0.52 (0.48, 0.56)
**Systolic blood pressure**		
Intercept	-0.26 (-0.29, -0.23)	-0.37 (-0.40, -0.33)
Age	-0.014 (-0.021, -0.007)	0.018 (0.009, 0.027)
Treatment for blood pressure	-0.22 (-0.26, -0.18)	-0.27 (-0.31, -0.23)
Number of repeats	0.053 (0.045, 0.060)	0.070 (0.059, 0.080)
Random effects variance	0.35 (0.33, 0.37)	0.32 (0.30, 0.34)
**Total cholesterol**		

	Coefficient (95% CI) Women	Coefficient (95% CI) Men
Intercept	0.14 (0.09, 0.19)	0.43 (0.37, 0.49)
Age	-0.040 (-0.051, -0.029)	0.009 (-0.005, 0.024)
Number of repeats	0.009 (-0.005, 0.023)	0.005 (-0.010, 0.021)
Random effects variance	0.74 (0.69, 0.80)	0.72 (0.66, 0.78)
**HDL cholesterol**		
Intercept	0.13 (0.05, 0.21)	0.08 (0.01, 0.15)
Age	-0.021 (-0.038, -0.003)	-0.001 (-0.019, 0.016)
Number of repeats	-0.011 (-0.032, 0.009)	-0.031 (-0.049, -0.012)
Random effects variance	1.24 (1.14, 1.35)	0.92 (0.84, 1.01)
**Landmark age 55**		
**Smoking**		
Intercept	0.22 (0.20, 0.24)	0.26 (0.23, 0.28)
Age	0.018 (0.013, 0.022)	0.010 (0.004, 0.015)
Number of repeats	0.005 (-0.000, 0.010)	0.004 (-0.003, 0.011)
Random effects variance	0.56 (0.53, 0.60)	0.50 (0.46, 0.54)
**Systolic blood pressure**		
Intercept	-0.07 (-0.10, -0.03)	-0.25 (-0.29, -0.21)
Age	-0.005 (-0.013, 0.002)	0.015 (0.006, 0.025)
Treatment for blood pressure	-0.20 (-0.24, -0.16)	-0.29 (-0.33, -0.25)
Number of repeats	0.054 (0.047, 0.062)	0.069 (0.059, 0.079)
Random effects variance	0.40 (0.38, 0.42)	0.33 (0.31, 0.36)
**Total cholesterol**		
Intercept	0.36 (0.31, 0.42)	0.43 (0.37, 0.49)
Age	-0.039 (-0.052, -0.026)	0.005 (-0.008, 0.018)
Number of repeats	0.011 (-0.002, 0.024)	0.001 (-0.014, 0.017)
Random effects variance	0.81 (0.74, 0.87)	0.74 (0.67, 0.81)
**HDL cholesterol**		
Intercept	0.24 (0.15, 0.33)	0.08 (0.00, 0.15)
Age	0.007 (-0.013, 0.026)	0.000 (-0.016, 0.016)
Number of repeats	-0.039 (-0.063, -0.016)	0.003 (-0.018, 0.024)
Random effects variance	1.34 (1.22, 1.46)	0.87 (0.79, 0.96)
**Landmark age 60**		
**Smoking**		
Intercept	0.22 (0.19, 0.24)	0.26 (0.23, 0.28)
Age	0.008 (0.004, 0.013)	0.013 (0.007, 0.018)
Number of repeats	0.001 (-0.005, 0.007)	-0.006 (-0.013, 0.001)
Random effects variance	0.51 (0.48, 0.55)	0.53 (0.49, 0.57)
**Systolic blood pressure**		
Intercept	0.07 (0.04, 0.11)	-0.07 (-0.11, -0.02)
Age	0.005 (-0.002, 0.013)	0.013 (0.004, 0.023)
Treatment for blood pressure	-0.20 (-0.24, -0.16)	-0.32 (-0.36, -0.28)
Number of repeats	0.054 (0.046, 0.062)	0.058 (0.048, 0.067)
Random effects variance	0.37 (0.35, 0.40)	0.38 (0.35, 0.41)
**Total cholesterol**		
Intercept	0.40 (0.34, 0.45)	0.37 (0.31, 0.43)
Age	-0.010 (-0.022, 0.002)	0.013 (0.000, 0.025)
Number of repeats	0.008 -(0.005, 0.021)	0.008 (-0.005, 0.021)
Random effects variance	0.82 (0.75, 0.88)	0.80 (0.73, 0.88)
**HDL cholesterol**		
Intercept	0.25 (0.17, 0.34)	0.15 (0.08, 0.23)
Age	-0.003 (-0.021, 0.015)	-0.009 (-0.026, 0.008)
Number of repeats	-0.029 (-0.051, -0.007)	-0.010 (-0.027, 0.006)
Random effects variance	1.32 (1.20, 1.45)	0.95 (0.85, 1.05)
**Landmark age 65**		
**Smoking**		
Intercept	0.17 (0.14, 0.19)	0.23 (0.20, 0.26)
Age	0.020 (0.016, 0.025)	0.014 (0.009, 0.019)
Number of repeats	-0.002 (-0.009, 0.004)	-0.008 (-0.016, 0.000)
Random effects variance	0.45 (0.42, 0.49)	0.47 (0.43, 0.52)
**Systolic blood pressure**		

	Coefficient (95% CI) Women	Coefficient (95% CI) Men
Intercept	0.14 (0.10, 0.18)	-0.04 (-0.09, 0.01)
Age	0.018 (0.010, 0.027)	0.025 (0.015, 0.035)
Treatment for blood pressure	-0.20 (-0.24, -0.16)	-0.30 (-0.34, -0.26)
Number of repeats	0.067 (0.058, 0.076)	0.025 (0.015, 0.035)
Random effects variance	0.33 (0.31, 0.36)	0.33 (0.31, 0.37)
**Total cholesterol**		
Intercept	0.44 (0.38, 0.50)	0.30 (0.24, 0.37)
Age	0.001 (-0.010, 0.013)	0.011 (-0.001, 0.022)
Number of repeats	-0.009 (-0.023, 0.005)	-0.022 (-0.039, -0.005)
Random effects variance	0.84 (0.77, 0.91)	0.75 (0.67, 0.82)
**HDL cholesterol**		
Intercept	0.28 (0.19, 0.36)	0.22 (0.12, 0.31)
Age	0.007 (-0.010, 0.023)	0.006 (-0.012, 0.023)
Number of repeats	-0.023 (-0.044, -0.003)	-0.017 (-0.043, 0.009)
Random effects variance	1.19 (1.08, 1.31)	0.95 (0.85, 1.07)
**Landmark age 70**		
**Smoking**		
Intercept	0.15 (0.12, 0.17)	0.17 (0.14, 0.20)
Age	0.012 (0.008, 0.017)	0.019 (0.013, 0.025)
Number of repeats	0.002 (-0.004, 0.008)	-0.001 (-0.010, 0.008)
Random effects variance	0.47 (0.43, 0.51)	0.49 (0.44, 0.54)
**Systolic blood pressure**		
Intercept	0.33 (0.28, 0.38)	0.05 (-0.01, 0.11)
Age	0.018 (0.008, 0.027)	0.040 (0.029, 0.052)
Treatment for blood pressure	-0.21 (-0.26, -0.17)	-0.35 (-0.40, -0.30)
Number of repeats	0.051 (0.042, 0.060)	0.058 (0.046, 0.070)
Random effects variance	0.32 (0.29, 0.35)	0.36 (0.32, 0.39)
**Total cholesterol**		
Intercept	0.35 (0.28, 0.43)	0.22 (0.14, 0.29)
Age	0.012 (0.000, 0.023)	0.020 (0.007, 0.034)
Number of repeats	0.000 (-0.017, 0.017)	-0.007 (-0.024, 0.009)
Random effects variance	0.77 (0.69, 0.85)	0.76 (0.68, 0.86)
**HDL cholesterol**		
Intercept	0.35 (0.25, 0.46)	0.23 (0.11, 0.35)
Age	-0.014 (-0.031, 0.003)	0.022 (-0.003, 0.046)
Number of repeats	-0.035 (-0.061, -0.009)	-0.022 (-0.050, 0.006)
Random effects variance	1.19 (1.06, 1.33)	1.08 (0.94, 1.24)
**Landmark age 75**		
**Smoking**		
Intercept	0.15 (0.12, 0.18)	0.13 (0.09, 0.16)
Age	0.006 (0.002, 0.011)	0.009 (0.003, 0.015)
Number of repeats	-0.006 (-0.013, 0.002)	-0.000 (-0.008, 0.007)
Random effects variance	0.47 (0.43, 0.52)	0.48 (0.43, 0.55)
**Systolic blood pressure**		
Intercept	0.49 (0.43, 0.54)	0.18 (0.11, 0.25)
Age	0.020 (0.009, 0.031)	0.031 (0.018, 0.044)
Treatment for blood pressure	-0.30 (-0.34, -0.25)	-0.32 (-0.38, -0.26)
Number of repeats	0.068 (0.056, 0.080)	0.045 (0.034, 0.057)
Random effects variance	0.36 (0.33, 0.39)	0.40 (0.36, 0.45)
**Total cholesterol**		
Intercept	0.30 (0.21, 0.38)	0.13 (0.04, 0.21)
Age	0.009 (-0.0204, 0.022)	0.013 (-0.002, 0.028)
Number of repeats	0.016 (-0.002, 0.035)	-0.004 (-0.020, 0.013)
Random effects variance	0.80 (0.71, 0.89)	0.73 (0.63, 0.84)
**HDL cholesterol**		
Intercept	0.34 (0.21, 0.47)	0.33 (0.19, 0.49)
Age	-0.009 (-0.031, 0.013)	-0.010 (-0.032, 0.012)
Number of repeats	-0.021 (-0.048, 0.006)	-0.016 (-0.048, 0.015)
Random effects variance	1.12 (0.97, 1.29)	1.18 (1.01, 1.38)

	Coefficient (95% CI) Women	Coefficient (95% CI) Men
**Landmark age 80**		
**Smoking**		
Intercept	0.10 (0.07, 0.13)	0.13 (0.09, 0.17)
Age	0.006 (0.001, 0.010)	0.008 (0.003, 0.013)
Number of repeats	0.001 (-0.006, 0.008)	-0.004 (-0.015, 0.007)
Random effects variance	0.50 (0.45, 0.56)	0.43 (0.37, 0.49)
**Systolic blood pressure**		
Intercept	0.53 (0.46, 0.60)	0.13 (0.04, 0.21)
Age	0.048 (0.037, 0.059)	0.044 (0.030, 0.058)
Treatment for blood pressure	-0.37 (-0.42, -0.32)	-0.29 (-0.36, -0.22)
Number of repeats	0.081 (0.066, 0.095)	0.072 (0.054, 0.090)
Random effects variance	0.41 (0.37, 0.45)	0.34 (0.30, 0.39)
**Total cholesterol**		
Intercept	0.32 (0.21, 0.42)	0.10 (-0.02, 0.22)
Age	0.040 (0.024, 0.057)	0.036 (0.019, 0.052)
Number of repeats	-0.007 (-0.030, 0.016)	-0.019 (-0.046, 0.009)
Random effects variance	0.97 (0.85, 1.11)	0.54 (0.45, 0.64)
**HDL cholesterol**		
Intercept	0.38 (0.21, 0.55)	0.34 (0.11, 0.57)
Age	0.009 (-0.007, 0.034)	0.003 (-0.025, 0.030)
Number of repeats	-0.038 (-0.076, -0.004)	-0.002 (-0.054, 0.050)
Random effects variance	1.19 (1.00, 1.41)	1.21 (0.99, 1.49)
**Landmark age 85**		
**Smoking**		
Intercept	0.07 (0.04, 0.11)	0.12 (0.07, 0.17)
Age	0.006 (0.001, 0.012)	0.002 (-0.003, 0.008)
Number of repeats	0.002 (-0.00, 0.002)	-0.008 (-0.023, 0.007)
Random effects variance	0.44 (0.39, 0.50)	0.54 (0.45, 0.64)
**Systolic blood pressure**		
Intercept	0.56 (0.48, 0.64)	0.01 (-0.12, 0.14)
Age	0.053 (0.040, 0.066)	0.095 (0.076, 0.115)
Treatment for blood pressure	-0.26 (-0.33, -0.20)	-0.34 (-0.44, -0.24)
Number of repeats	0.047 (0.032, 0.062)	0.077 (0.041, 0.113)
Random effects variance	0.37 (0.33, 0.42)	0.47 (0.39, 0.56)
**Total cholesterol**		
Intercept	0.19 (0.09, 0.30)	-0.06 (-0.21, 0.09)
Age	0.042 (0.023, 0.062)	0.048 (0.025, 0.071)
Number of repeats	0.015 (-0.004, 0.034)	-0.001 (-0.041, 0.040)
Random effects variance	0.87 (0.74, 1.02)	0.74(0.59, 0.92)
**HDL cholesterol**		
Intercept	0.41 (0.23, 0.58)	0.11 (-0.10, 0.32)
Age	-0.017 (-0.049, 0.016)	0.003 (-0.026, 0.033)
Number of repeats	-0.024 (-0.052, 0.004)	0.002 (-0.054, 0.058)
Random effects variance	1.32 (1.08, 1.60)	0.75 (0.57, 0.97)

HDL = high-density lipoprotein.

Web Table 5: Hazard ratios from the Cox proportional hazards models in the study sample

	HR (95% CI)
**Basic model**	
Landmark age	1.14 (1.10, 1.18)
Landmark age squared	1.00 (1.00, 1.00)
Diabetes status	1.31 (1.13, 1.53)
Treatment for blood pressure	1.27 (1.15, 1.39)
**Estimated current values model**	
Landmark age	1.12 (1.08, 1.16)
Landmark age squared	1.00 (1.00, 1.00)
Diabetes status	1.40 (1.20, 1.64)
Treatment for blood pressure	1.10 (0.99, 1.23)
Smoking status	2.16 (1.90, 2.45)
Systolic blood pressure^	1.36 (1.25, 1.48)
Total cholesterol	1.31 (1.19, 1.43)
HDL cholesterol	0.89 (0.80, 0.98)

HDL = high-density lipoprotein.

*HRs are given per standard deviation (SD) increase (SD = 21.97 for systolic blood pressure, SD = 1.20 for total cholesterol and SD = 0.40 for HDL cholesterol).

^Adjusted for treatment for hypertension.

Web Table 6: Hazard ratios from the Cox proportional hazards models in the restricted sample

	HR (95% CI)
**Basic model**	
Landmark age	1.08 (1.00, 1.16)
Landmark age squared	1.00 (1.00, 1.00)
Diabetes status	1.23 (0.96, 1.59)
Treatment for blood pressure	1.16 (0.96, 1.40)
**Last observation carried forward model**	
Landmark age	1.08 (1.00, 1.16)
Landmark age squared	1.00 (1.00, 1.00)
Diabetes status	1.30 (1.00, 1.69)
Treatment for blood pressure	1.19 (0.99, 1.45)
Smoking status	1.94 (1.57, 2.39)
Systolic blood pressure	1.12 (1.00, 1.26)
Total cholesterol	1.19 (1.07, 1.32)
HDL cholesterol	0.90 (0.82, 1.00)
**Cumulative means model**	
Landmark age	1.07 (0.99, 1.15)
Landmark age squared	1.00 (1.00, 1.00)
Diabetes status	1.34 (1.03, 1.74)
Treatment for blood pressure	1.13 (0.92, 1.38)
Smoking status	1.94 (1.56, 2.42)
Systolic blood pressure	1.20 (1.04, 1.38)
Total cholesterol	1.23 (1.10, 1.38)
HDL cholesterol	0.90 (0.81, 1.00)
**Estimated current values model**	
Landmark age	1.07 (0.99, 1.15)
Landmark age squared	1.00 (1.00, 1.00)
Diabetes status	1.35 (1.04, 1.76)
Treatment for blood pressure	1.08 (0.87, 1.34)
Smoking status	2.15 (1.68, 2.75)
Systolic blood pressure^	1.23 (1.04, 1.47)
Total cholesterol	1.29 (1.13, 1.48)
HDL cholesterol	0.89 (0.79, 1.00)

HDL = high-density lipoprotein.

*HRs are given per standard deviation (SD) increase (SD = 21.97 for systolic blood pressure, SD = 1.20 for total cholesterol and SD = 0.40 for HDL cholesterol).

^Adjusted for treatment for hypertension.

Web Figure 3: Calibration plots for each risk prediction model using the full data set

a. Estimated current values of risk factors

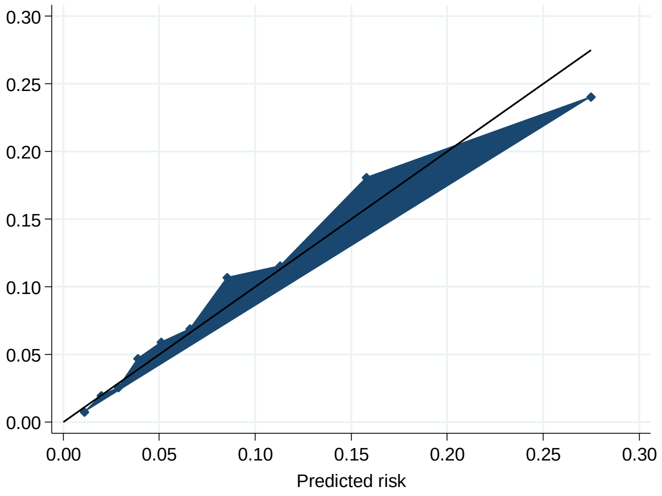

b. Estimated current values of risk factors with age interactions

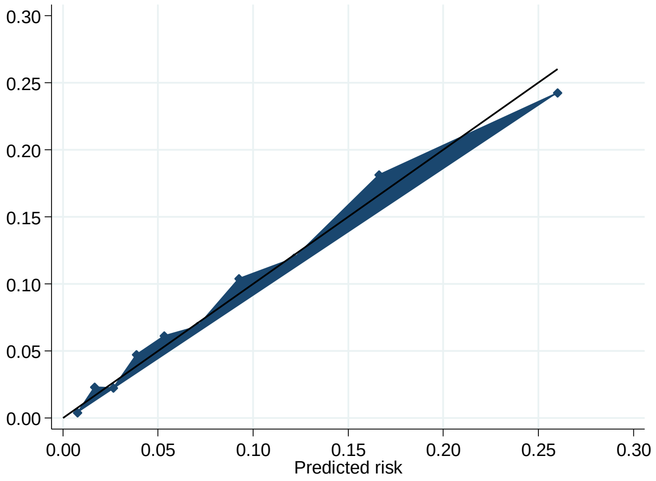

c. Basic model

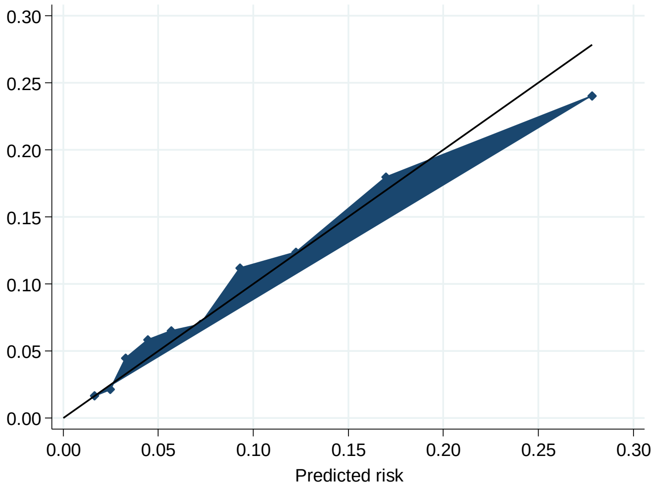

Web Figure 4: Overall and age-adjusted C-index across landmark ages—basic model

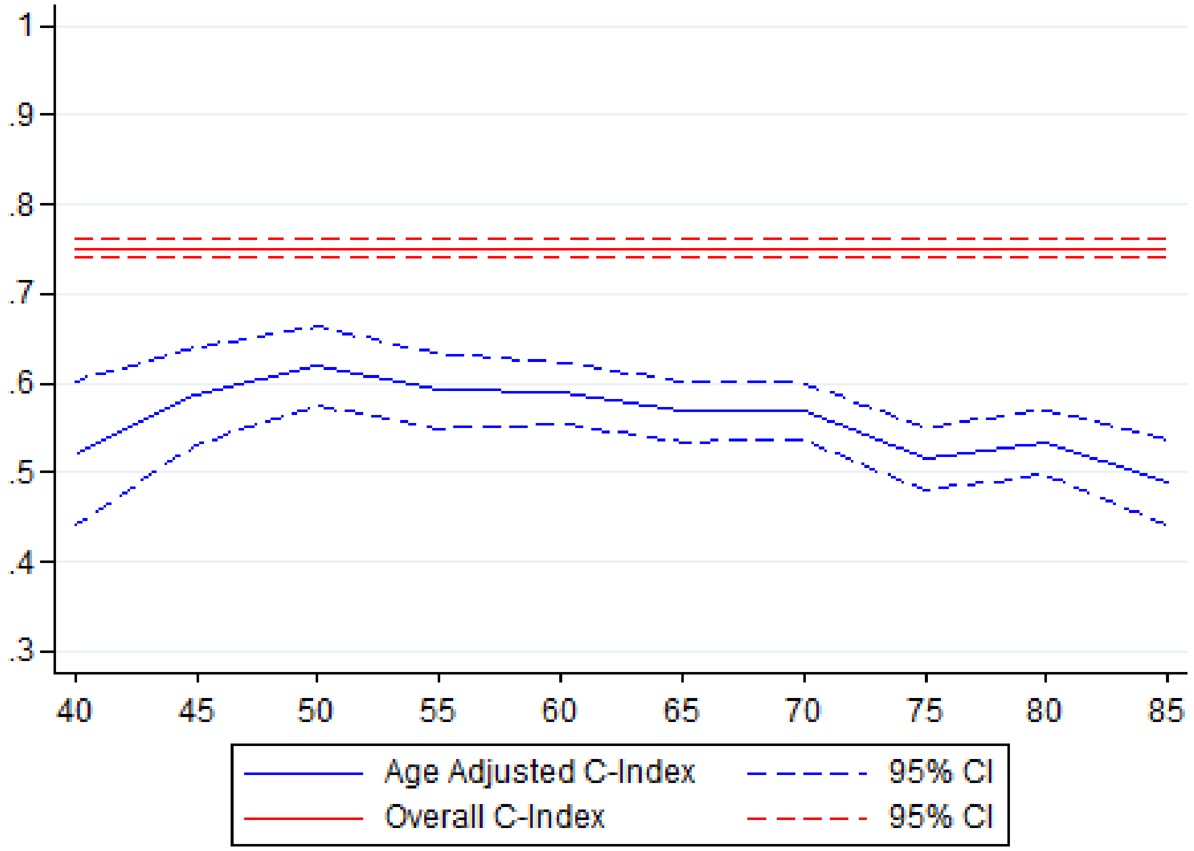

Web Figure 5: Distribution of absolute cardiovascular disease risk scores in the validation samples for each risk prediction model in the restricted data set

Last observed risk factor values

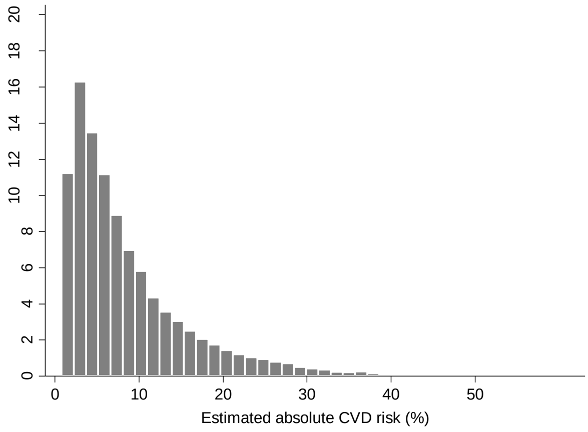

Cumulative means of risk factors

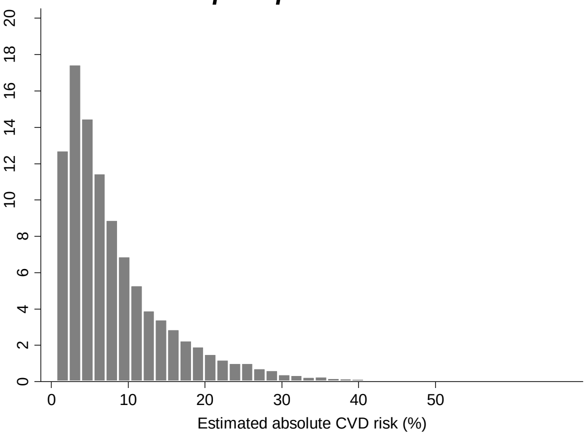

Estimated current values of risk factors

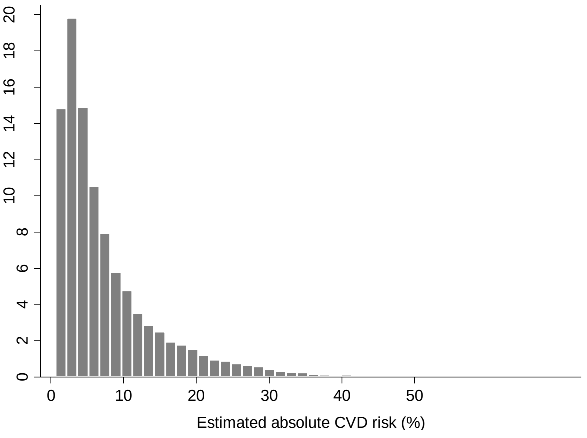

Among individuals in the restricted sample, the percentage of individuals with an estimated 10-year CVD risk of <10%, 10-19% and ≥20% were 70%, 21% and 9% for the last observed risk factor model, 71%, 20% and 9% for the cumulative means model and 72%, 20% and 9% for the estimated current values model.
In the study sample, the percentage of individuals with an estimated 10-year CVD risk of <10%, 10-19% and ≥20% was 72%, 16% and 11% for the basic model and 73%, 17% and 10% for the estimated current values model.

Web Figure 6: Calibration plots for each risk prediction model using the restricted data set

Last observed risk factor values

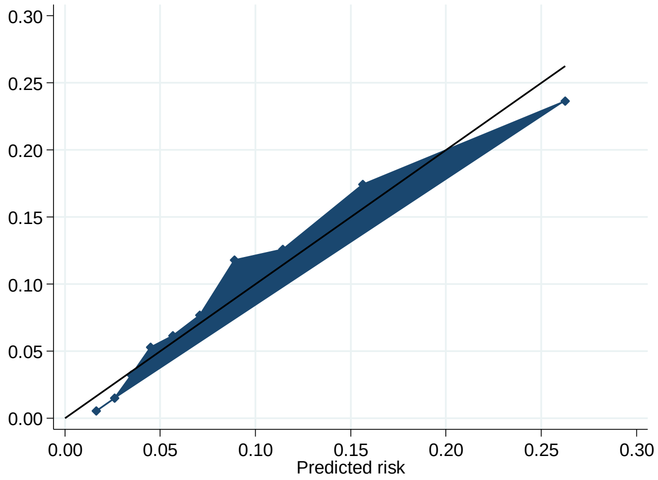

Last observed risk factor values with age interactions

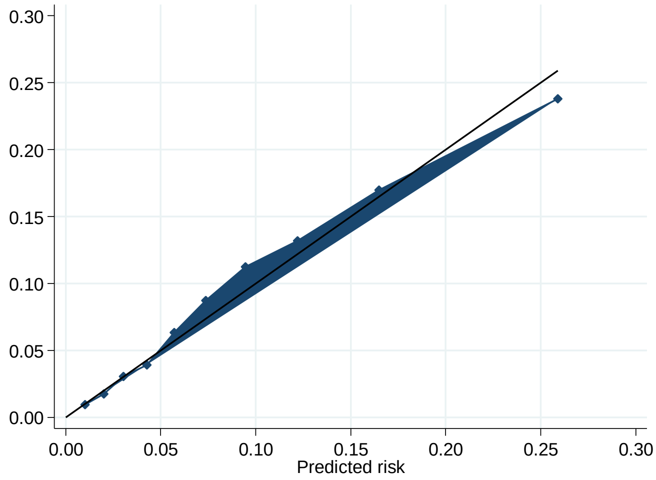

Cumulative means of risk factors

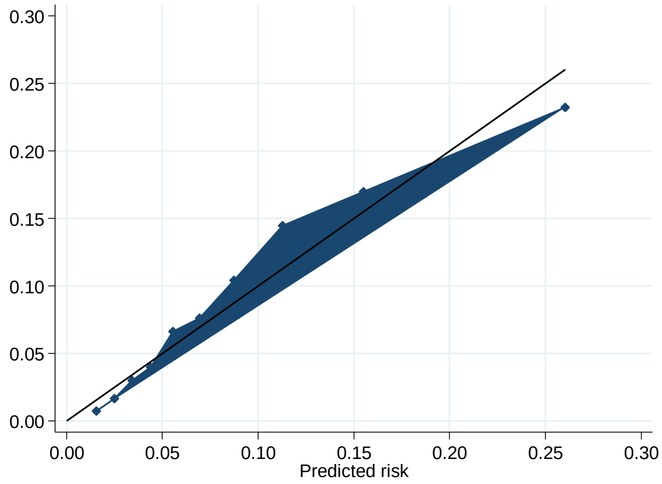

Cumulative means of risk factors with age interactions

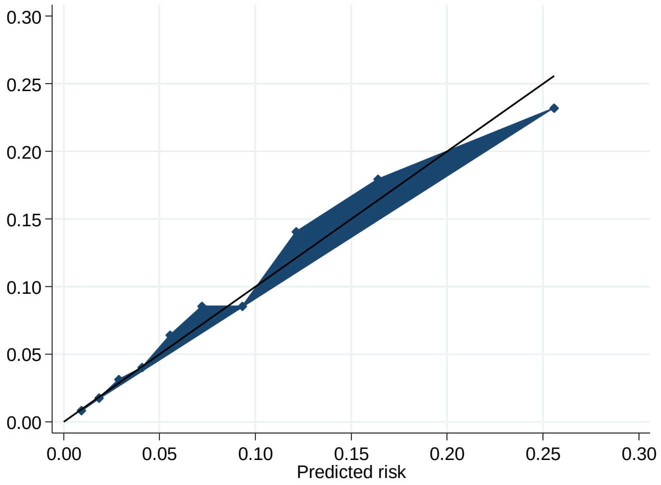

Estimated current values of risk factors

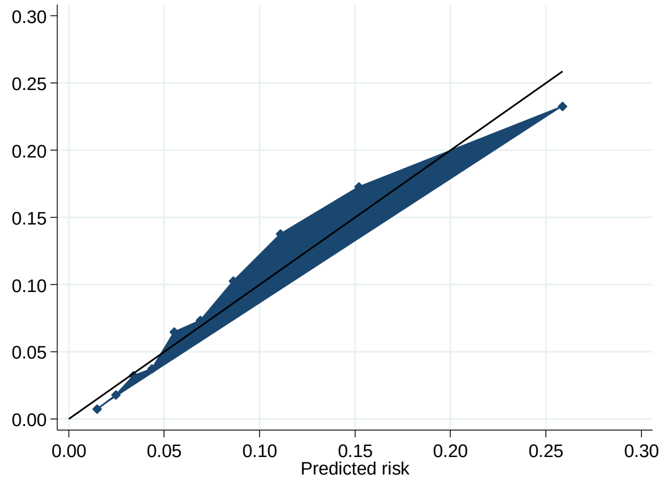

Estimated current values of risk factors with age interactions

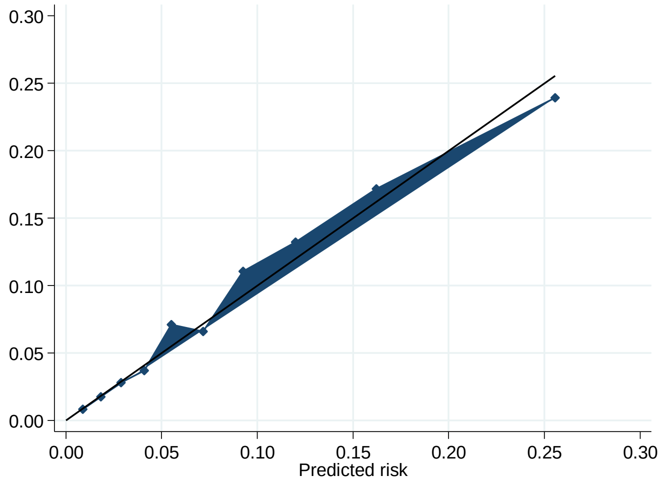

Web Figure 7: Calibration and risk discrimination statistics for each risk prediction model in the restricted sample* (*n* = 12,292)

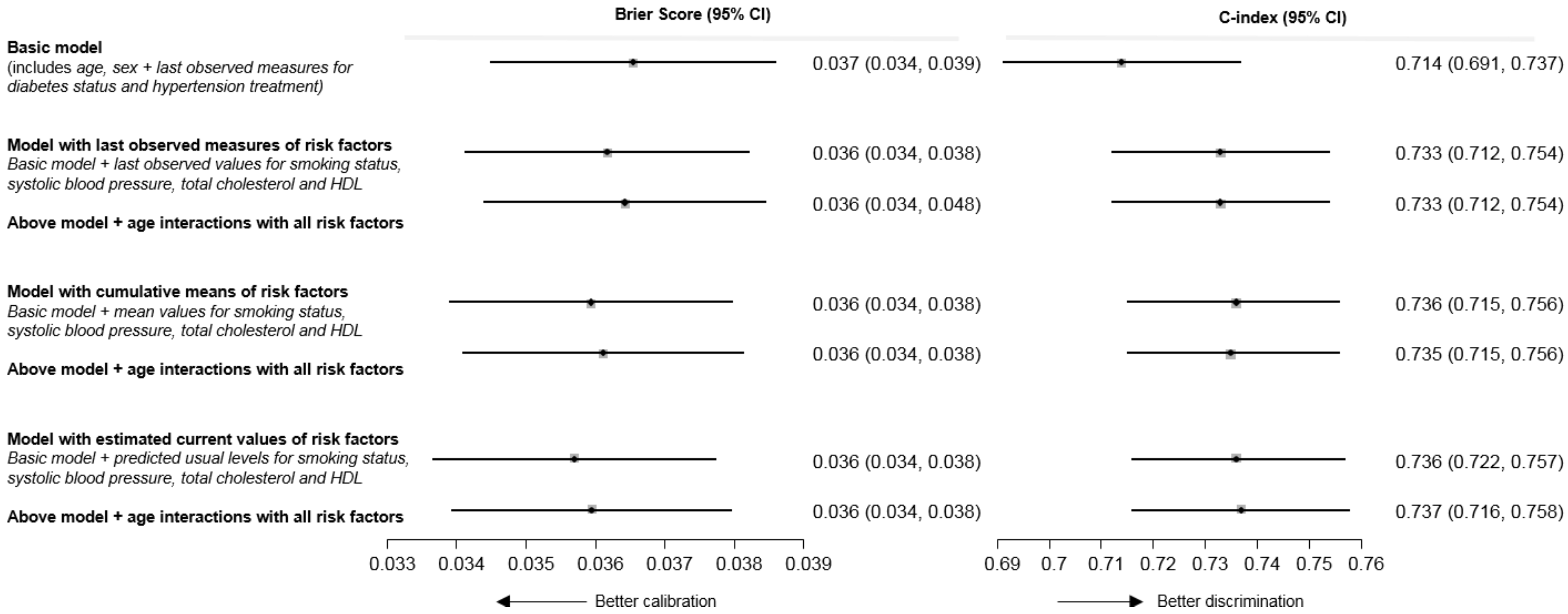

CI = confidence interval.
*Restricted sample contains only patients with at least one measurement of each smoking status, systolic blood pressure, total cholesterol and HDL-C.

Web Figure 8: Overall and age-adjusted C-index across landmark age models in the restricted sample

Basic model

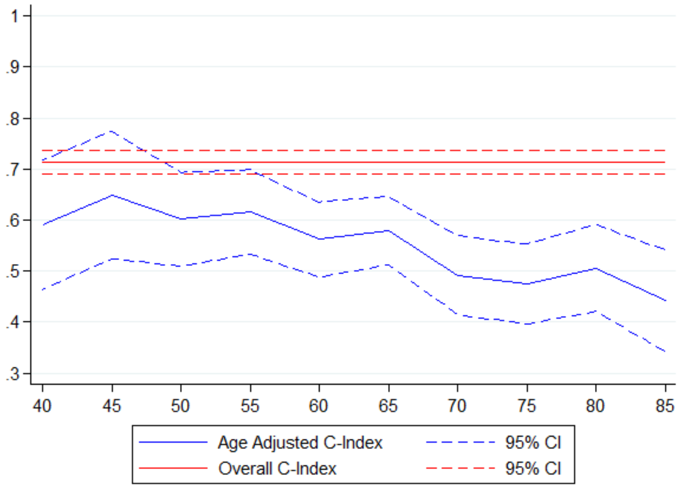

Last observed risk factor values

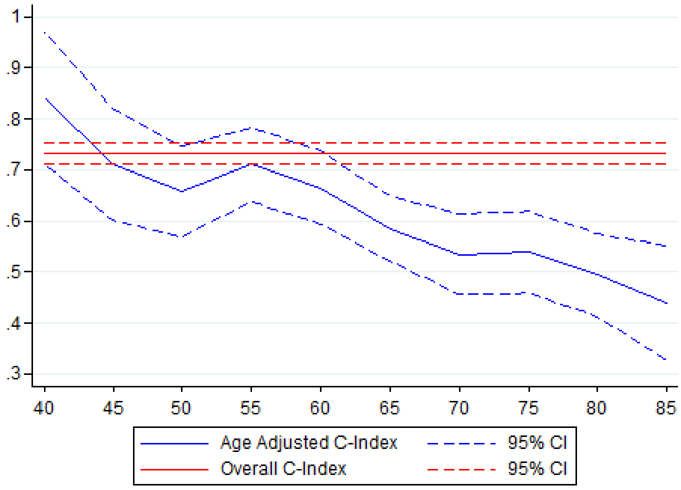

Last observed risk factor values with age interactions

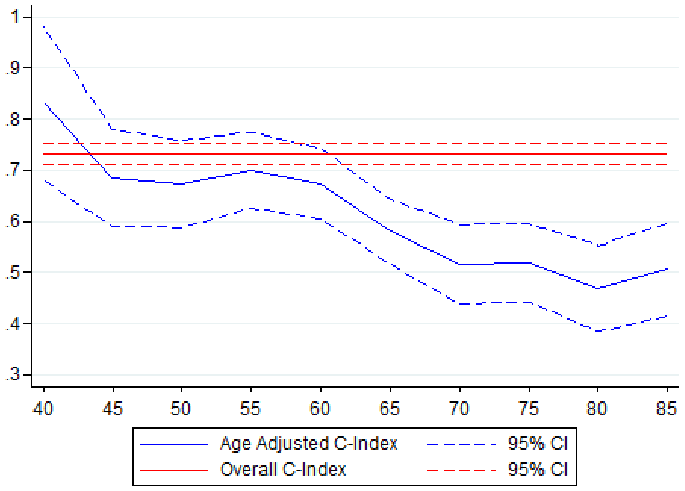

Cumulative means of risk factors

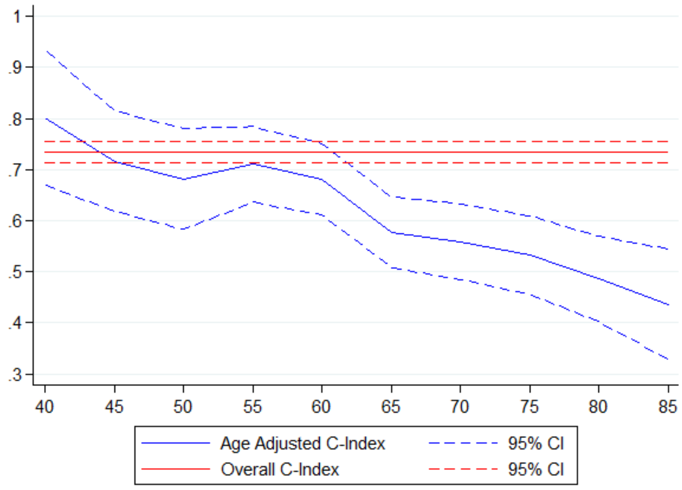

Cumulative means of risk factors with age interactions

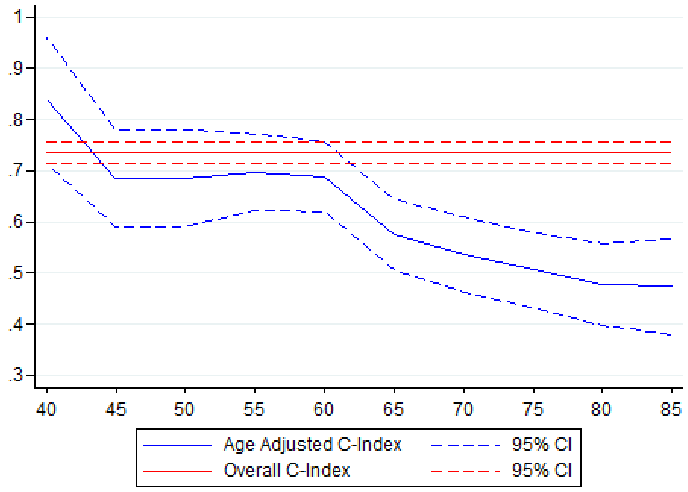

Estimated current values of risk factors

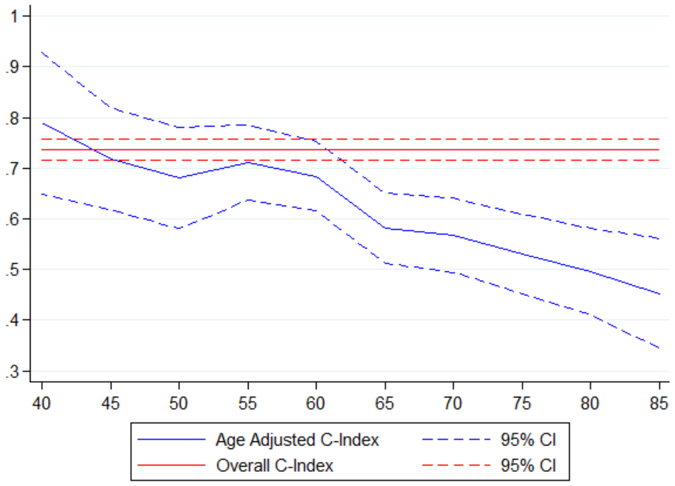

Estimated current values of risk factors with age interactions

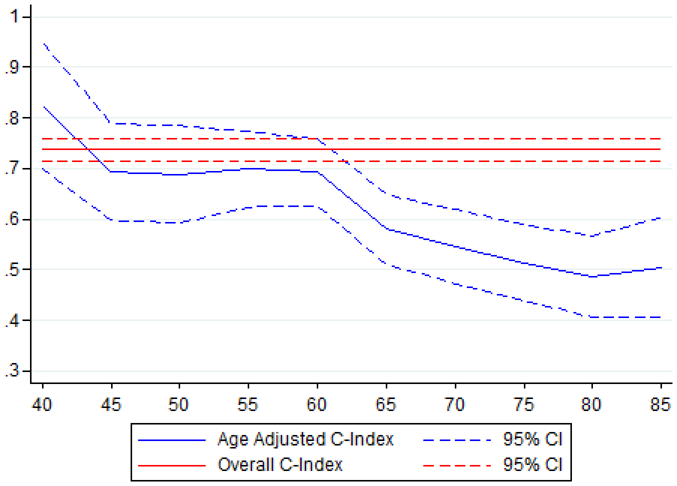

Web Table 7. Calibration and risk discrimination statistics for each risk prediction model in the study sample with statin users included (*n* = 36,297)

Risk Factors Included	Brier Score (95% CI)	C-Index (95% CI)
Basic model<br>Age, sex + last observed measures for diabetes status and hypertension treatment	0.045 (0.044, 0.046)	0.735 (0.726, 0.743)
Estimated current levels<br>Above + predicted usual levels for smoking status, systolic blood pressure, total cholesterol and HDL-C	0.045 (0.044, 0.046)	0.751 (0.743, 0.760)
Above + age-interactions with all risk factors	0.046 (0.045, 0.047)	0.753 (0.744, 0.761)

CI = confidence interval; HDL-C = high-density lipoprotein cholesterol.

Note: lower Brier score = better calibration; higher C-index = greater risk discrimination.

Web Table 8. Calibration and risk discrimination statistics for each risk prediction model in the restricted sample* with statin users included (*n* = 18,054)

Risk Factors Included	Brier Score (95% CI)	C-Index (95% CI)
Basic model<br>Age, sex + last observed measures for diabetes status and hypertension treatment	0.046 (0.044, 0.048)	0.686 (0.670, 0.702)
Last observations values<br>Basic model + last observed values for smoking status, systolic blood pressure, total cholesterol and HDL-C	0.046 (0.044, 0.048)	0.703 (0.688, 0.719)
Above + age-interactions with all risk factors	0.045 (0.044, 0.047)	0.704 (0.688, 0.719)
Cumulative means<br>Basic model + mean values for smoking status, systolic blood pressure, total cholesterol and HDL-C	0.045 (0.044, 0.047)	0.705 (0.690, 0.721)
Above + age-interactions with all risk factors	0.045 (0.043, 0.047)	0.705 (0.690, 0.721)
Estimated current levels<br>Basic model + predicted usual values for smoking status, systolic blood pressure, total cholesterol and HDL-C	0.045 (0.044, 0.047)	0.707 (0.692, 0.723)
Above + age-interactions with all risk factors	0.046 (0.045, 0.048)	0.708 (0.693, 0.723)

CI = confidence interval; HDL-C = high-density lipoprotein cholesterol.

*Restricted sample contains only patients with at least one measurement of each smoking status, systolic blood pressure, total cholesterol and HDL-C.

Note: lower Brier score = better calibration; higher C-index = greater risk discrimination.
